# Unveiling the role of epigenetics in leaf senescence: a comparative study to identify different epigenetic regulations of senescence types in barley leaves

**DOI:** 10.1186/s12870-024-05573-9

**Published:** 2024-09-14

**Authors:** Elżbieta Rudy, Umesh Kumar Tanwar, Zofia Szlachtowska, Magda Grabsztunowicz, Magdalena Arasimowicz-Jelonek, Ewa Sobieszczuk-Nowicka

**Affiliations:** 1 Department of Plant Physiology, Faculty of Biology, Adam Mickiewicz University, Uniwersytetu Poznańskiego 6 Str., Poznań, 61-614 Poland; 2 Department of Plant Ecophysiology, Faculty of Biology, Adam Mickiewicz University, Uniwersytetu Poznańskiego 6 Str., Poznań, 61-614 Poland

**Keywords:** Barley, Leaf senescence, Histone modifiers, DNA modifiers, ATP-dependent chromatin remodelers

## Abstract

**Background:**

Developmental leaf senescence (DLS) is an irreversible process followed by cell death. Dark-induced leaf senescence (DILS) is a reversible process that allows adaptations to changing environmental conditions. As a result of exposure to adverse environmental changes, plants have developed mechanisms that enable them to survive. One of these is the redirection of metabolism into the senescence pathway. The plant seeks to optimise resource allocation. Our research aims to demonstrate how epigenetic machinery regulates leaf senescence, including its irreversibility.

**Results:**

*In silico* analyses allowed the complex identification and characterisation of 117 genes involved in epigenetic processes in barley. These genes include those responsible for DNA methylation, post-translational histone modifications, and ATP-dependent chromatin remodelling complexes. We then performed RNAseq analysis after DILS and DLS to evaluate their expression in senescence-dependent leaf metabolism. Principal component analysis revealed that evaluated gene expression in developmental senescence was similar to controls, while induced senescence displayed a distinct profile. Western blot experiments revealed that senescence engages senescence-specific histone modification. During DILS and DLS, the methylation of histone proteins H3K4me3 and H3K9me2 increased. H3K9ac acetylation levels significantly decreased during DILS and remained unchanged during DLS.

**Conclusions:**

The study identified different epigenetic regulations of senescence types in barley leaves. These findings are valuable for exploring epigenetic regulation of senescence-related molecular mechanisms, particularly in response to premature, induced leaf senescence. Based on the results, we suggest the presence of an epigenetically regulated molecular switch between cell survival and cell death in DILS, highlighting an epigenetically driven cell survival metabolic response.

**Supplementary Information:**

The online version contains supplementary material available at 10.1186/s12870-024-05573-9.

## Background

Plants have the unique challenge of being unable to move away from adverse environmental conditions. Some mechanisms have evolved to cope with this problem. One of them is called senescence. In these cases, recycling nutrients becomes a critical concern whenever possible. From a physiological perspective, the current understanding of senescence supports the following definition: (i) Senescence is a developmental phase that occurs as a temporary differentiation at the end of growth. (ii) It may or may not be followed by death. (iii) Senescence is entirely dependent on the viability of cells and the expression of specific genes [1, 2]. Genetic interventions have shown that death does not necessarily require senescence, nor is it an inevitable consequence of it [1].

Dark-induced senescence has been used experimentally as an easy way to study the progress of leaf senescence. However, detailed studies of gene expression patterns have revealed discrepancies between the dark-induced and developmentally controlled processes [3]. Dark-induced leaf senescence (DILS) results in an apparent loss of chlorophyll, disassembly of cellular elements and a lack of photosynthetic activity, none of which can be distinguished from the age-dependent natural senescence. However, the lack of coordinated cell development within a single leaf introduces complexity in the leaf senescence study. Thus, induced senescence, which directs a synchronous process, like dark-induced senescence, has become relevant [4–7]. It also eliminates misleading factors that coincide with developmental senescence, such as bolting or flowering [8]. As the course of the senescence process is related to plant species, plant developmental stage, and plant environmental conditions, these treatments cannot be considered the same. The genome resources available for *Arabidopsis* have made it a useful model of identification and functional analysis of genes regulated by senescence [9–11]. However, in many plants, such as barley, the removal of developing flowers and pods significantly extends the life of their leaves, while in *Arabidopsis*, male-sterile mutants from which developing bolts have been removed do not extend the life of leaves. Because of these differences, cereal leaves must be used as an equivalent to the *Arabidopsis* model for leaf senescence studies in cereal [3]. Apparent differences in the senescence program of *Arabidopsis* compared to monocotyledonous plants were found. The senescence in cereals is generally regulated at the single-leaf level. Nutrients from older leaves are remobilised for younger leaves and ultimately for the flag leaf, thus contributing to the nutrients necessary for the development of the grain. Cereal leaves have a meristem base, the leaf tip consists of older cells, and younger ones are concentrated at the base of the leaf. This cell organisation makes it easier to differentiate the progression of senescence [8]. Among cereals, barley is a model organism from a genetic and genomic point of view. Barley is characterised by a high degree of natural variation and its adaptability to several different cultivation environments. Its diploid genome, whose sequence is available, the self-pollinating mating system and the availability of genetic and genomic resources make this plant a reference model [12].

Law et al. [7] propose a model illustrating the specific metabolic strategies employed by leaves in response to two darkening treatments in *Arabidopsis*: an individually darkened leaf - which supports rapid senescence - and a leaf from a whole darkened plant - characterised by a strong capacity for survival. Several external stimuli, different from darkness, can also induce the onset and progression of senescence or processes resembling senescence and share some common pathways [13]. These include climate change-related environmental stresses, such as temperature extremes, water stress, nutrient deficiencies, or light conditions. It is likely that the recycling of nutrients is a crucial concern wherever possible, thus resembling senescence, with the possible exception when the speed of the response to changing conditions is of greater importance to the plant as a whole [1]. Biotic and abiotic stresses significantly reduce barley yield in many parts of the world [14, 15]. Environmental stresses can severely affect its development and yield in the juvenile stages of wheat, from sowing to tillering. Such pre- and post-harvest losses can amount to as much as 30% of the crop [16].

Epigenetic and epigenomic studies of regulating gene expression to specific stress and the origin of this specificity in crops are still unknown. Genetic modifications have been used for crop improvement; however, using epigenetic modifications is at its beginning. We developed a barley crop model for early and late events during dark-induced leaf senescence referred to in the manuscript as the DILS program, to examine induced leaf senescence. The idea of a program as applied to living systems has been taken from computer science. The system is built in a particular way, so it always starts and fails in more or less the same manner. Sobieszczuk-Nowicka et al. [12] presented transcriptomic, cytological, and physiological data that revealed events in the barley DILS program, differences from developmental senescence, the time limit for dark-to-light transition for reversal of the senescence process, and progression of senescence through autophagy into the PCD phase.

We also showed the most evident differences in gene medleys between DILS and developmental leaf senescence (DLS), including DNA modifications active only in DILS [12].

Moreover, our preliminary study using large-scale *Arabidopsis* expression data reported by Breeze et al. [11] and available barley leaf senescence-related microarrays [12, 17, 4], we determined the transcription pattern during senescence of genes which are either known or suggested to be involved in plant DNA methylation processes. Genes such as AGO10 (which encodes a member of the elongation initiation factor and plays a central role in RNA silencing processes as essential components of the RNA-induced silencing complex), methyltransferase 1 (MET1) and repressor of silencing 1 (ROS1) were significantly regulated at the transcription level in DILS. In developmental leaf senescence opposite to DILS MET1 and ROS1 transcript levels decreased before the leaves were fully expanded (reviewed in [18]).

This suggested the possibility of a yet-to-be-discovered epigenetic-based switch between cell survival and cell death in the DILS program. As a control, DLS was used as an irreversible process followed by cell death.

With this in mind, as most of the epigenetic regulatory genes in barley were not identified, we focus first on identifying them at a whole genome scale. In silico analyses allowed the first identification of 117 genes involved in epigenetic processes in barley and their characteristics. Further, we verify whether gene regulation of these pathways includes differential changes and whether this control level is implemented by different but interacting and often interdependent epigenetic mechanisms, including DNA methylation, covalent histone modifications, and non-covalent chromatin remodelling that may allow rapid response to signalling and stimuli when the expression of a new proteins needs to be adjusted rapidly. Indeed, induced senescence and its reversal include the sudden change from anabolism to catabolism and inverse, which contrasts with the subtle shift that occurs when the process occurs naturally, steering the expression of senescence-associated genes – SAGs [12].

Understanding the mechanisms of epigenetic regulators and their regulatory networks in this process in crops may be a potential tool for further exploitation toward sustainable agriculture (so-called epi-breeding) [19]. Moreover, designing new breeding strategies that consider epigenetic variability is desirable. This seems even more realistic with the advancement of genomic technologies and the cost-lowering of next-generation sequencing. Like marker-assisted selection, epigenetic marker-assisted selection could also be initiated.

## Results

### Identification and characterisation of epigenetic regulators in barley

In this study, 117 genes were identified as the epigenetic regulatory genes in barley (*HvERG*s), divided into three major classes: histone modifiers, DNA modifiers, and ATP-dependent chromatin remodelers (Additional file 1: Table S1). Histone modifiers included seven histone acetyltransferases (*HATs*), 17 histone deacetylases (*HDAC*s), 26 histone methyltransferases (*HMT*s), and 14 histone demethylases (*HDM*s). *HATs* consisted of two *HAG*s (*HvHAG1-2*), one *HAM* (*HvHAM*), three *HAC*s (*HvHAC1-3*), and one *HAF* (*HvHAF1*). *HDAC*s included 12 *HDA*s (*HvHDA1-3*,* HvHDA5-8*,* HvHDA9-14*), two *SRT*s (*HvSRT1-2*), and three *HDT*s (*HvHDT1-3*). *HMT*s were composed of 25 *SDG*s (*HvSDG1-26*) and one *PRMT* (*HvPRMT1*). *HDMs* included four *HDMAs* (*HvHDMA1-4*) and 11 *JMJs* (*HvJMJ1-11*). DNA modifiers included 12 DNA methyltransferases (*DNMT*s) and three DNA demethylases (*DME*s). *DNMT*s consisted of three *MET*s (*HvMET1-3*), three *CMT*s (*HvCMT1-3*), five *DRM*s (*HvDRM1-5*), and one *DNMT* (*HvDNMT2*). DNA demethylases included three *DME*s (*HvDME1-3*). ATP-dependent chromatin remodelers included 13 *Snf2-like* (*HvSnf2_1–3*,* HvLsh_1–3*,* HvIswi_1–2*,* HvALC1*,* HvChd1 and HvMi-2_1–3*), three *Swr1-like* (*HvSwr1*,* HvIno80*,* HvEtl1*), eight *Rad54-like* (*HvRad54*,* HvATRX. HvDRD1_1–6*), eight *Rad5/16-like* (*HvRad5_16_1–4*,* HvRis1_1–3*,* HvSHPRH*), five *SSO1653-like* (*HvMot1*,* HvERCC6_1–4)*,* and* one *SMARCAL1-like (HvSMARCAL1_1*) genes. A brief characterisation of the proteins of identified genes has been performed (Additional file 1: Table S2).

**Fig. 1:**
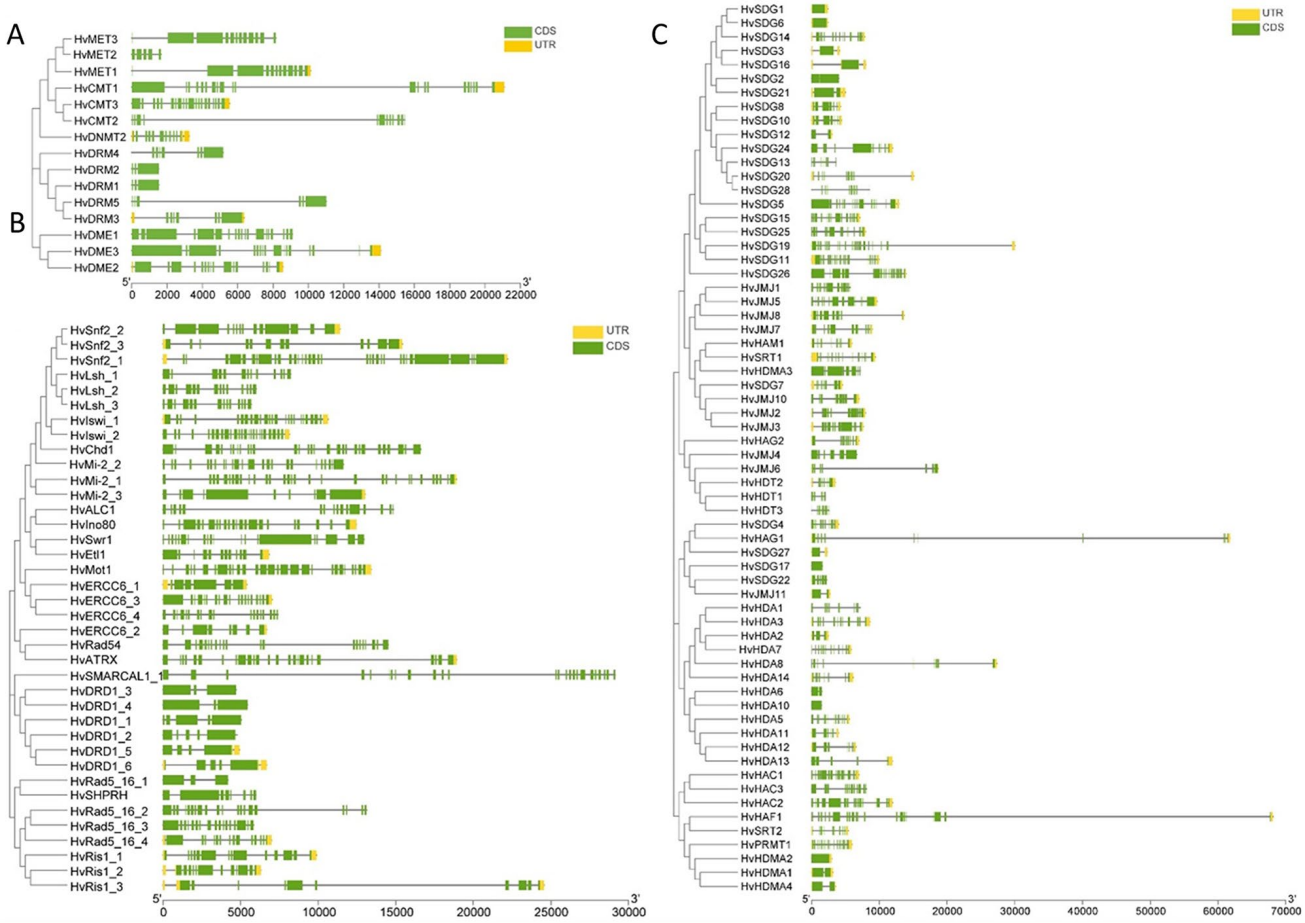
Gene structure showing the intron-exon organisation of epigenetic regulatory genes in barley. (**A**) DNA modifiers, (**B**) ATP-dependent chromatin remodelers, and (**C**) histone modifiers. The scale bar presents gene length in base pairs

The gene structure analysis revealed that all the DNA modifiers genes and ATP-dependent chromatin remodelers genes were composed of multiple exons ranging from three (*HvDRM1*,*2*) to 21 (*HvCMT1*) in DNA modifiers, three (*HvDRD1_3* and *HvDRD1_4*) to 34 (*HvSnf2_1*) in the ATP-dependent chromatin remodelers (Fig. 1A, B). However, the histone modifiers contained single exon genes (*HvHDA10*,* HvSDG1-3*,*-6*,*-16*,*-17*,* -27*,* HvHDMA2*) and up to 22 exons (*HvHAF1*) (Fig. 1C). The protein sequences analysis showed the distribution of ten conserved motifs and ten different domain families in proteins encoded by DNA modifiers genes (Additional file 2: Fig. S1A). In contrast, ten conserved motifs and 23 families of specific domains were found within proteins encoded by ATP-dependent chromatin remodelers genes (Additional file 2: Fig. S1B**).** Among proteins encoded by histone modifier genes, ten motifs and 50 specific domains were identified (Additional file 2: Fig. S2). The phylogenetic trees were constructed to get evolutionary insight into epigenetic regulatory genes in barley and other plant species. The results indicate that most barley proteins clustered with corresponding orthologs in *O. sativa* and *S. bicolor* (Additional file 2: Fig S3-S4). With meticulous attention to detail, we compared the barley epigenetic regulatory genes with the genomes of *O. sativa*,* S. bicolor*,* B. distachyon*, and *A. thaliana* (Additional file 2: Fig. S5, Additional file 1: Table S3). In the DNA modifiers, three, four, and five genes were in synteny with *S. bicolor*,* O. sativa*, and *B. distachyon*, respectively. In contrast, no gene was found in the syntenic homology with *A. thaliana*. Among ATP-dependent chromatin remodelers, 23 and 26 genes appeared to have syntenic homology with *S. bicolor* and *B. distachyon*, respectively, while only two were syntenic with *O. sativa* and *A. thaliana*. In the histone modifiers, 37, 43, and 39 genes were in synteny with *S. bicolor*,* O. sativa*, and *B. distachyon*, respectively, while only one gene was found in the syntenic homology with *A. thaliana*.

**Fig. 2:**
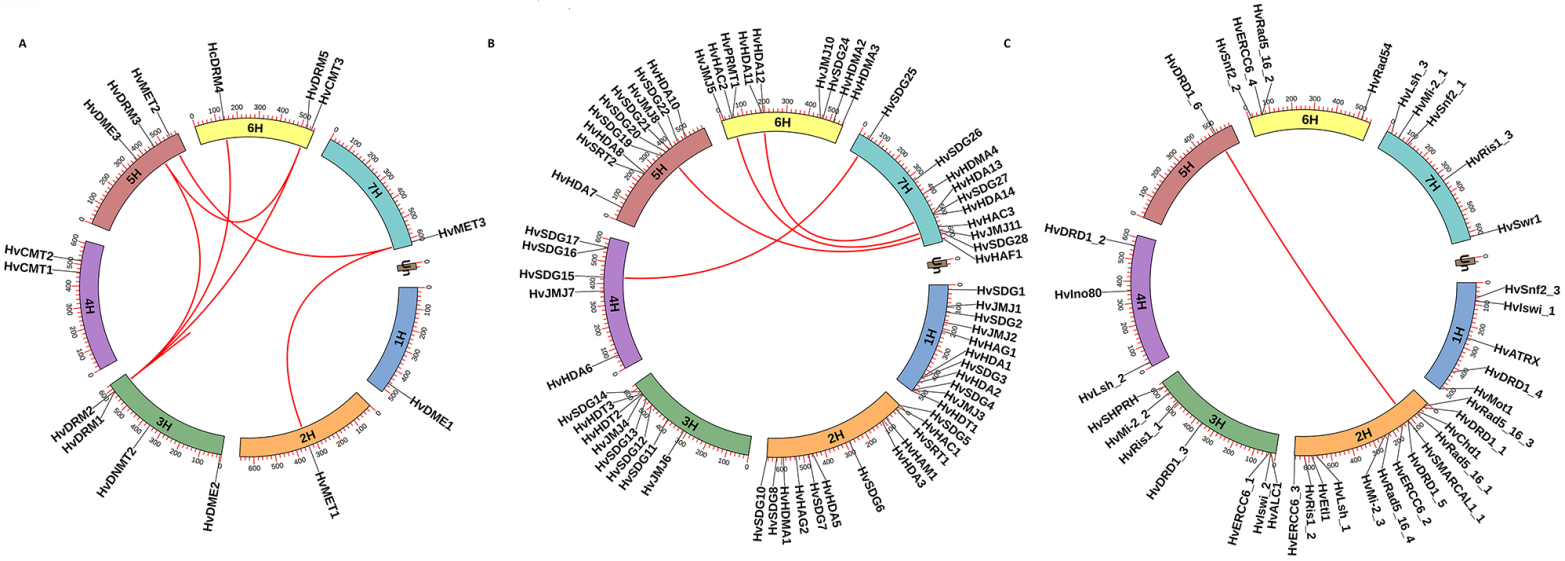
Chromosomal distribution and gene duplication of epigenetic regulatory genes in barley. (**A**) DNA modifiers, (**B**) histone modifiers, (**C**) ATP-dependent chromatin remodelers. The duplicated gene pairs are linked with a red line

Our analysis of the chromosomal location of the identified genes revealed a distinct pattern. All the epigenetic regulatory genes were found to be randomly distributed on all seven chromosomes in the barley genome (Fig. 2). However, for DNA modifiers, chromosomes 1H, 2H, 7H were less representative and contained only one gene each (*HvDME1*, *HvMET1* and *HvMET3*, respectively) (Fig. 2A), and for ATP-dependent chromatin remodelers, chromosome 5H contained only one gene (*HvDRD1_6*) (Fig. 2C). This distribution pattern could potentially have profound implications for our understanding of barley’s epigenetic regulation. Gene duplication analysis further deepened our insights. Among histone modifiers, one gene pair (*HvHAC2/HvHAC3*) in *HATs*, one gene pair (*HvHDA12/HvHDA13*) in *HDACs*, two gene pairs (*HvSDG25/HvSDG15* and *HvSDG28/HvSDG20*) in *HMTs* were found to be duplicated (Fig. 2B). Among DNA modifiers, seven gene pairs (*HvMET1/HvMET3*,* HvMET3/HvMET2*, *HvDRM5/HvDRM3*,* HvDRM2/HvDRM1*,* HvDRM2/HvDRM5*,* HvDRM2/HvDRM3*, and *HvDRM2/HvDRM4*) in *DNMTs* had duplication. In comparison, only one gene pair (*HvDRD1_5/HvDRD1_6*) in ATP-dependent chromatin remodelers had duplication event in barley genome (Fig. 2C). The Ka/Ks values for the duplicated gene pairs were in the range of 0.13 (*HvSDG28/HvSDG20*) to 0.19 (*HvHAC2/HvHAC3*) for histone modifiers, 0.10 (*HvMET3/HvMET2*) to 0.47 (*HvDRM2/HvDRM4*) for DNA modifiers. They were 0.37 (*HvDRD1_5/HvDRD1_6*) for the ATP-dependent chromatin remodelers (Additional file 1: Table S4). These values provide crucial insights into the evolutionary dynamics of these genes. Furthermore, the divergence time for the duplicated genes was estimated to be in the range of 15.3 (*HvSDG28/HvSDG20*) to 74.8 (*HvHAC2/HvHAC3*) MYA for histone modifiers, 29.3 (*HvDRM2/HvDRM1*) to 76.3 (*HvMET1/HvMET3*) MYA for DNA modifiers, and 25.5 (*HvDRD1_5/HvDRD1_6*) MYA for ATP-dependent chromatin remodelers (Additional file 1: Table S4). These divergence times provide a fascinating glimpse into the evolutionary history of these genes.

### Promoter analysis, microRNA target site, and protein-protein interaction predictions

Next, we analysed the promoter regions of epigenetic regulatory genes. The identification of *cis*-acting regulatory elements (CREs) resulted in the CREs being divided into four categories: growth and development, stress response, light response, and hormone response (Additional File 1: Table S5). CAAT-box and TATA-box represented the growth and development of CREs and were the most abundant CREs in all the epigenetic regulatory genes. In the stress-responsive CREs, STRE, MYB, and MYC elements were the most abundant among all the epigenetic regulatory genes. In the hormone-responsive CREs, ABREs were the most abundant and overrepresented by *DNMTs* in DNA modifiers, *HDMs* and *HDACs* in histone modifiers, and a few ATP-dependent chromatin remodelers. In the light-responsive CREs, G-box and G-Box elements were the prominent representatives in DNA and histone modifiers, and interestingly, no major CREs were found in the ATP-dependent chromatin remodelers. These CREs were overrepresented in *DNMT*s among DNA modifiers, and *HDACs*,* HMT*s, and *HDM*s among histone modifiers.

The analysis of transcription factor binding sites (TFbs) resulted in the identification of several TFbs, which were categorised into nine common TFbs (Additional File 2: Fig. S6). The prominent representatives of TFbs were TCP and AP2/ERF TFbs, followed by bZIP, MYB, GATA, bHLH, and WRKY in all the epigenetic regulatory genes. The least representative TFbs were NAC and BES1. Interestingly, WRKY TFbs were not found in the promoter regions of *HvHAC2* (*HAT*s), *HvHDA1*, and *HvHDA13* (*HDAC*s) in histone modifiers, and *HvEtl1*,* HvChd1*, and *HvATRX* in the ATP-dependent chromatin remodelers. Further, the promoter regions were analysed for CpG/CpNpG islands and tandem repeats. The CpG/CpNpG islands were identified in the promoter regions of four *HvDNMT*s, two *HvDME*s, six *HvHA*Ts, 13 *HvHDAC*s, 11 *HvHDM*s, and 29 ATP-dependent chromatin remodelers (Additional file 1: Table S6). Tandem repeats were identified in the promoter regions of one *HvDNMTs*, two *HvDME*s, four *HvHAT*s, nine *HvHDAC*s, four *HvHDM*s, and 12 ATP-dependent chromatin remodelers (Additional file 1: Table S7).

The coding sequences (CDS) of epigenetic genes were also analysed for the presence of miRNA targeting sites (Additional file 1: Table S8). The maximum number of targeting sites for miRNAs (37) were detected in the 27 ATP-dependent chromatin remodelers genes, and most of these genes were classified as *snf2-like* subfamily. In the histone modifiers, 23 miRNAs had targeting sites in 18 *HMT* genes, 19 in 10 *HDMs*, 12 in 11 *HDAC*s, and six in four *HAT*s. Among DNA modifiers, nine DNA methyltransferases contained targeting sites for 19 miRNAs, and three genes of DNA demethylases had targeting sites for seven miRNAs. The protein-protein interactions (PPI) network was further analysed for the barley epigenetic regulatory genes. The genes showed a considerable PPI within and between the groups (Additional file 2: Fig. S7).

### Expression of the epigenetic regulatory genes is changed in response to abiotic stresses

**Fig. 3:**
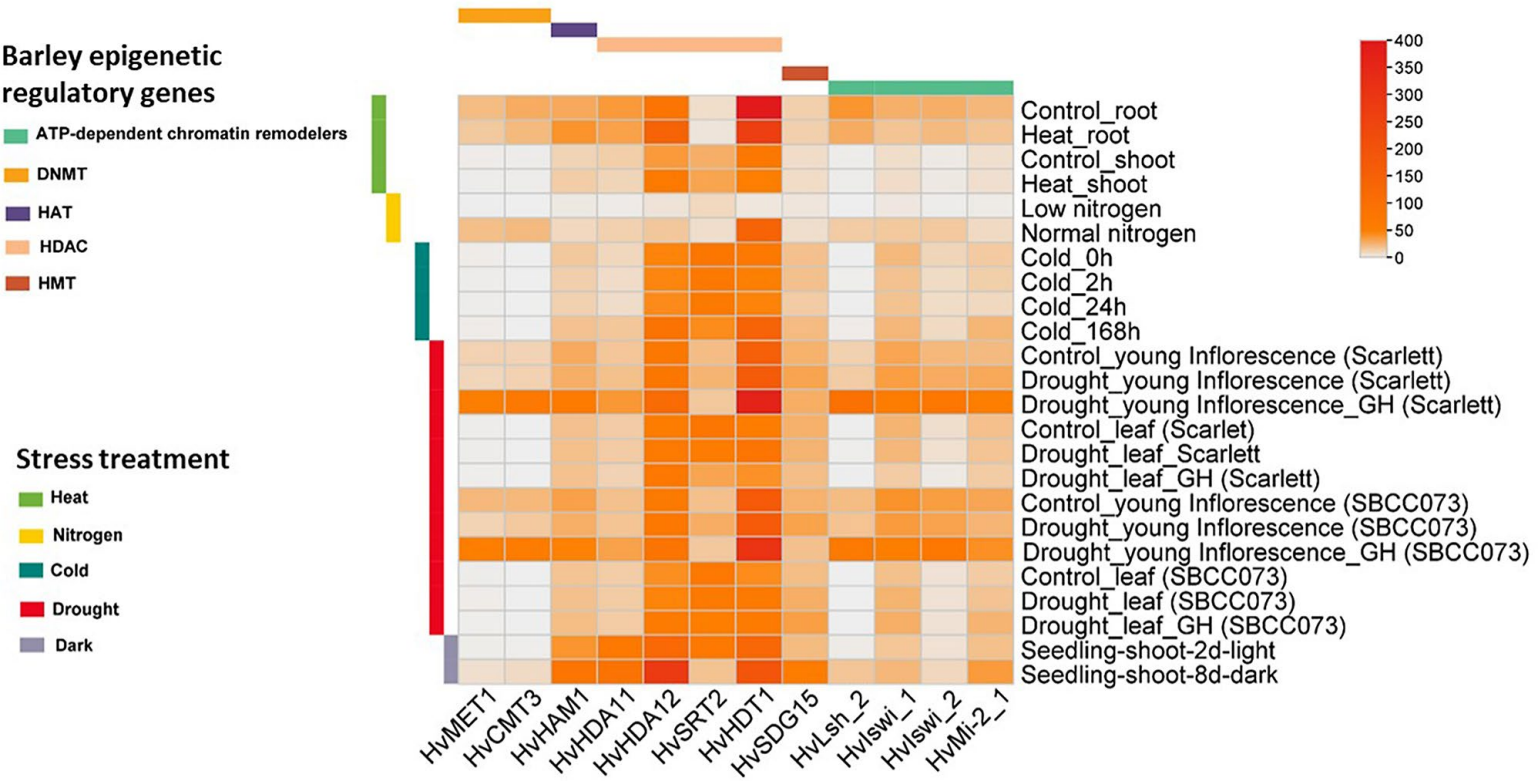
Expression patterns of epigenetic regulatory genes under various abiotic stresses, based on RNA-seq data from BarleyExpDb. The colour scale bar represents FPKM values in control and treated samples. Genes with expression levels exceeding 50 FPKM in at least one of the presented conditions were selected. HAT- histone acetyl transferase; HDAC- histone deacetylase; HMT- histone methyl transferase; DNMT- DNA methyl transferase. The detailed expression patterns of epigenetic regulatory genes under various abiotic stresses are available in Supplementary Material (Additional File 1: Table S9)

A publicly available database was used to analyse the expression of *HvERGs* in different abiotic stresses depicting various climatic conditions like cold, heat, waterlogging, and drought conditions (Fig. 3, Additional File 1: Table S9). Among histone modifiers, *HvHDT1* showed higher expression under drought, dark, and cold stress, while *HvHDA12* had higher expression under heat and dark stress treatments. Both *HvHAM1* and *HvHDA11* exhibited increased expression during drought stress. *HvSRT2* exhibited significantly reduced expression under dark stress compared to light conditions. Among ATP-dependent chromatin remodelers, *HvLsh2*,* HvIswi1*, *HvIswi2*, and *HvMi_2 − 1* showed higher expression under drought and dark stress. In the DNA modifiers, *HvCMT3* and *HvMET1* exhibited high expression in young inflorescence tissues under drought conditions (Fig. 3).

### Expression of the epigenetic regulatory genes is changed in response to different types of senescence

**Fig. 4:**
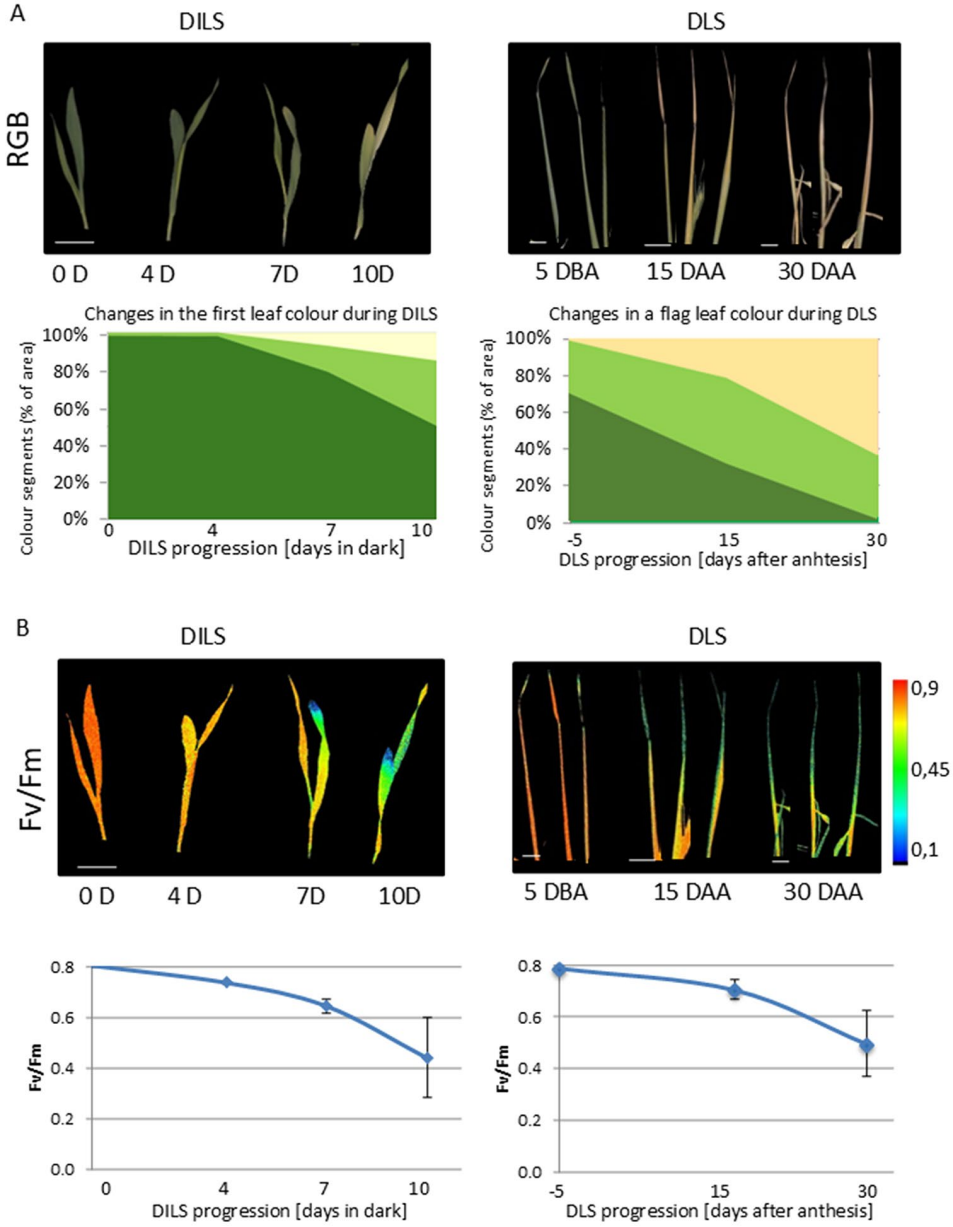
Experimental models. Changes in barley leaves phenotypes during DILS and DLS progression. (**A**) Monitoring senescence by RGB imaging. Top: spatiotemporal greenness heterogeneity in senescent seedlings and flag leaves. Bottom, changes in colour participation during senescence progression include dark green, light green, and yellow colours. (**B**) Fluorescence quenching analysis of senescence. On top are false colour images of sample plants showing spatiotemporal patterns of the maximum efficiency of primary photochemistry (Fv/Fm) on barley seedlings and flag leaves. The colour scale encoding fluorometric values is given next to the pictures. Bottom, the fluorescence emission values represent averages obtained from at least ten plants. Other parameters describing changes in the photosynthetic activity of plants are shown in Additional file 2: Fig. S9. 0D – control plants, D4- day 4 in dark; D7- day 7 in dark, D10- day 10 in dark, 5 DBA- 5 days before anthesis, 15 DAA- 15 days after anthesis, 30 DAA- 30 days after anthesis

To study the role of epigenetic mechanisms during leaf senescence regulation, senescence symptoms were analysed in two distinct models: young barley seedlings exposed to DILS and flag leaves during DLS (Fig. 4). Induced senescence leads to a minor decrease in the greenness level limited to the tip of the primary leaves where the oldest cells are located. During DLS, all flag leaves were gradually yellowing (Fig. 4A). The described loss of chlorophyll was followed by the gradual decrease in photosynthesis efficiency across the entire flag leaves exposed to DLS and in the primary leaves of seedlings exposed to DILS (Fig. 4B, Additional file 2: Fig. S8).

**Fig. 5:**
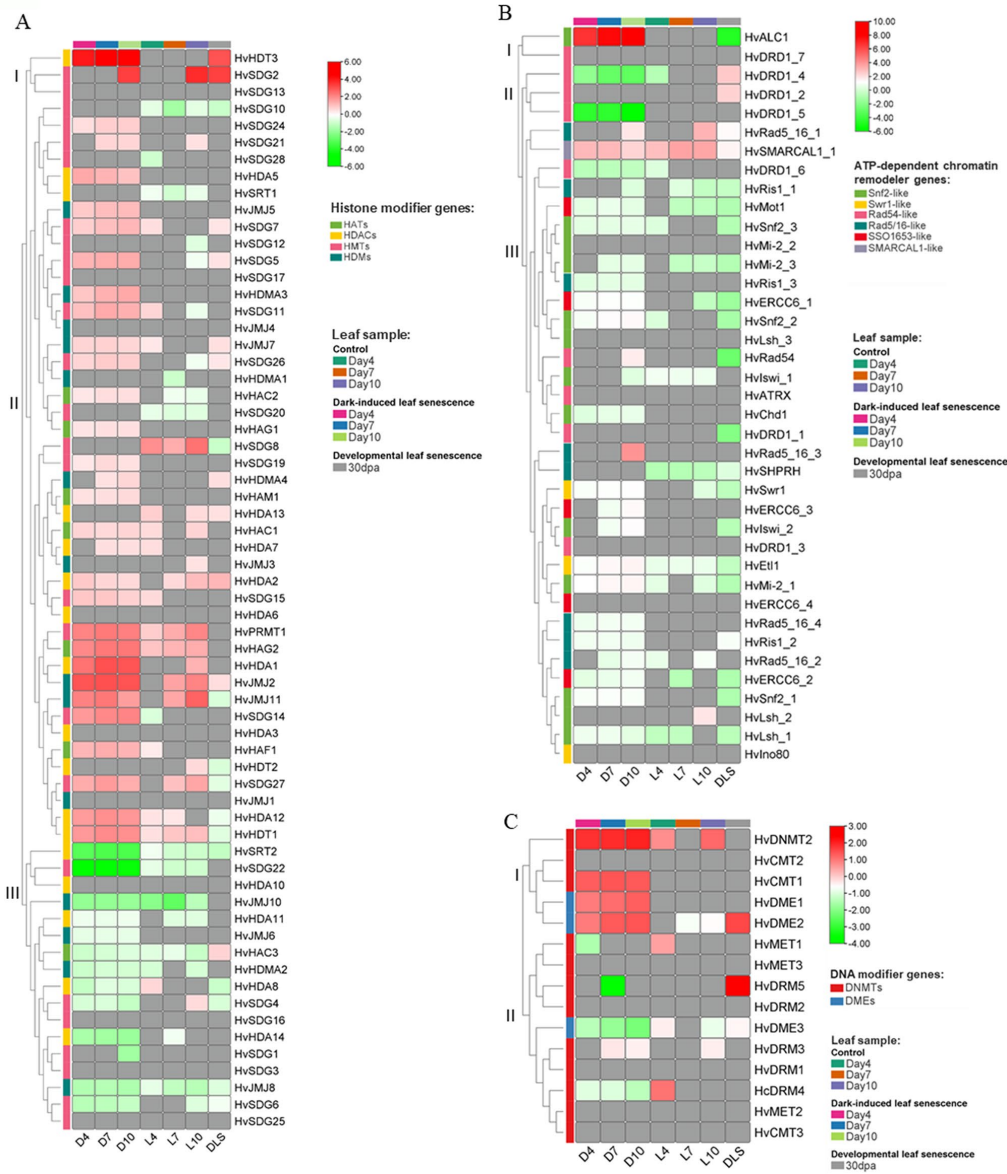
Heat map showing the expression profiles of barley epigenetic regulatory genes. (**A**) histone modifiers genes, (**B**) ATP-dependent chromatin remodeler genes, (**C**) DNA modifier genes during DILS and DLS. D4- day 4 in dark; D7- day 7 in dark, D10- day 10 in dark, L4- day 4 in light; L7- day 7 in light; L10- day 10 in light; DLS- 30 days post anthesis. For DILS, the gene expression was determined in barley first leaf compared to control plants (plants at day 0), and for DLS, barley flag leaf as compared to control (leaf 5 days prior- anthesis). HATs- histone acetyl transferases; HDACs- histone deacetylases; HMTs- histone methyl transferases; HDMs- histone demethylases; DNMTs- DNA methyl transferases; DMEs- DNA demethylases. The colour scale bar represents log 2 FC values. The grey colour shows expression below the limit of detection

Gene expression analysis was performed on three groups of investigated *HvERGs* during DILS and DLS (Fig. 5). The histone modifiers were grouped in three clusters based on the expressions profile (Fig. 5A). *HvHDT3* and *HvSDG2*, from cluster I, showed very high expression in DILS and DLS as well. From cluster II, upregulation of several histone lysine methyltransferases (*HvSDG5*,* HvSDG14*,* HvSDG27*, *HvSDG11)*, histone demethylase (*HvJMJ2* and *HvJMJ11*), protein arginine methyltransferase *HvPRMT1*, histone acetyltransferase *HvHAG2*, as well as histone deacetylases (*HvHDA1*,* HvHDA12*,* HvHvHDT1*) was observed during DILS. In Cluster III, downregulation of genes encoding histone lysine methyltransferases (*HvSDG4*,* HvSDG6*, *HvSDG22*), histone deacetylases (*HvHDMA2*,* HvHDA8*,* HvHDA11*,* HvHDA14 HvSRT2*) and histone demethylases (*HvJMJ6*,* HvJMJ8*,* HvJMJ10*) were observed in DILS.

The expression profile of ATP-dependent chromatin remodelers is divided into three clusters (Fig. 5B). From cluster I, the gene *HvALC1* from the *Snf2-like* group was highly expressed during DILS. *HvSMARCAL1* gene from cluster II showed increased expression in DILS and light conditions. Notably, the genes related to *Rad54-like* from cluster II, namely *HvDRD1_4*,* HvDRD1_5*, and *HvDRD1-6*, exhibited downregulation, highlighting the distinct epigenetic reprogramming events associated with DILS. Genes from cluster III, *HvDRD1_1*,* HvRad54*,* HvSnf2_2*,* and HvSnf2_1* were downregulated in DLS. An interesting observation was seen in the *HvALC1* remodeler gene, which showed upregulation during DILS and noticeable downregulation during DLS.

The analysis of gene expression levels revealed upregulation of DNA methyltransferases in cluster I, *HvCMT1*,* HvDNMT2*, and demethylases *HvDME1-2* during DILS (Fig. 5C). Conversely, in cluster II, downregulation was observed for methyltransferase *HvDRM5* on day 7 of DILS. In contrast, its expression level was high during DLS. Methyltransferase *HvDRM4* and demethylase *HvDME*3 were also downregulated during DILS. A notable difference in expression was observed for methyltransferase *HvMET1*, which was downregulated during DILS but upregulated under light conditions.

### Principal component analysis of the epigenetic regulatory genes’ expression profiles confirms inherent differences in gene expression patterns between different senescence types

**Fig. 6:**
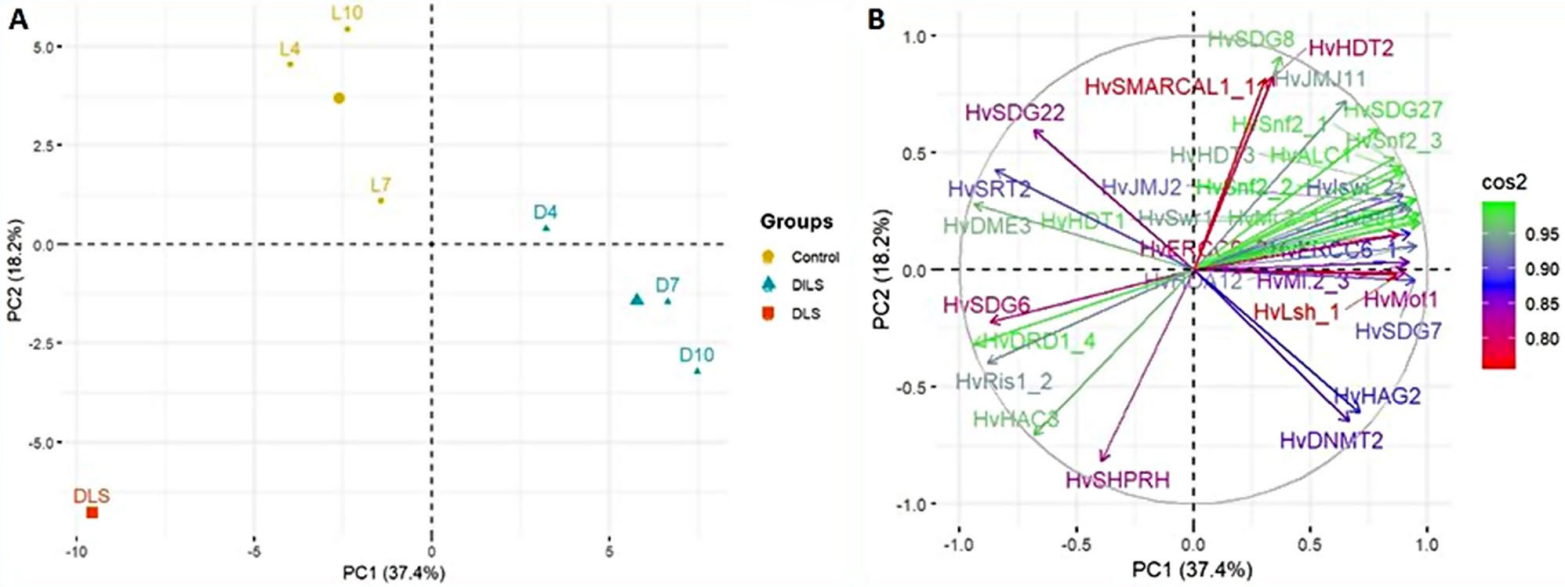
Principal component analysis of epigenetic regulatory genes based on the first two principal components. (**A**) PCA of individuals (samples). (**B**) PCA of variables (genes). L4- day 4 in light; L7- day 7 in light; L10- day 10 in light; D4- day 4 in dark; D7- day 7 in dark, D10- day 10 in dark; DLS- 30 days post anthesis. Sample groups are highlighted in yellow, blue, and red colours. The factor map helps to visualise the cluster of correlated variables in groups. Cos2 is the gradient of quality that highlights the most important variables in explaining the variations retained by the principal components

The study utilised Principal Component Analysis (PCA) as a robust analytical tool to recognise the functional implications of gene expression patterns obtained from our experimental dataset. PCA on the first two components explained 55.6% variability (PC1:37.4% and PC2:18.2%) and divided the studied samples into three main groups: Control (light), DILS, and DLS (Fig. 6A). Most of the evaluated genes showed cos2 > 0.5 for the PC1 and PC1 (Fig. 6B). The segregation of these clusters implies inherent differences in gene expression dynamics among the experimental conditions under investigation. The Control group was differentiated mainly by histone methyltransferase *HvSDG22*, histone acetyltransferases *HvHAC2*, *HvHAG2*, and DNA demethylase *HvDME*2 genes. Group DILS was distinguished by histone methyltransferase *HvSDG22*, histone acetyltransferases *HvHAG2*,* HvHAC2*, histone deacetylases *HvHDA12*,* HvSRT2*, DNA demethylase *HvDME3* and ATP-dependent chromatin remodelers *HvLsh_1*,* HvMot1*, *Hv ERCC6_2.* The DLS group was defined by histone methyltransferases *HvSDG8*,* HvSDG27*, histone demethylase *HvJMJ11*, histone acetyltransferase *HvHAC3*, histone deacetylase *HvHDT3*, and ATP-dependent chromatin remodelers *HvSHPRH*,* HvRis1_2*,* HvDRD1_4*,* HvSMARCAL1_1*,* HvSnf2_1*,* HvSnf2_2*,* HvSnf2_3*,* HvALC1.*

### Expression patterns of the epigenetic regulatory genes are significantly correlated during dark-induced leaf senescence but not in the control conditions

**Fig. 7:**
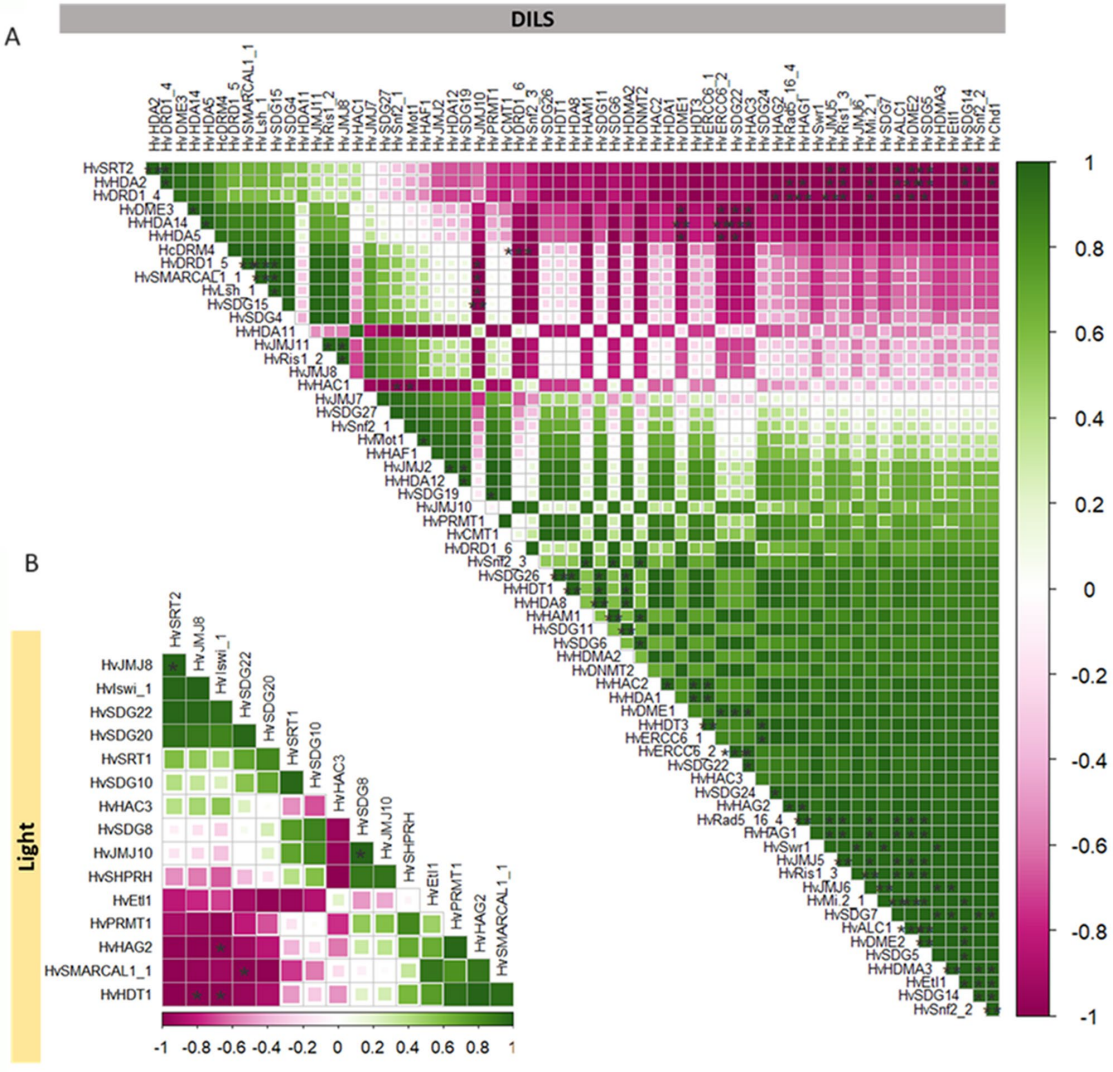
Pairwise correlations of differentially expressed epigenetic regulatory genes; Upper right panel (**A**) in DILS and lower left panel (**B**) in the control (light) condition. The genes are grouped as the first principal component order. The scale from green to purple shows the positive and negative correlation. Statistical significance is shown as *** *P* < 0.001, **P < 0.01, *P < 0.05

Further, we did the pairwise correlation analysis for the differentially expressed epigenetic regulatory genes in DILS and control (Light) samples. Our findings established a significant correlation in the expression patterns of *HvERG*s during DILS (Fig. 7A). At the same time, very few genes were significantly correlated in the control conditions (Fig. 7B). In general, histone deacetylases showed a negative correlation with histone methyl transferases and ATP-dependent chromatin remodelers. However, the other group of genes had a positive correlation. Among *HMT*s, a positive correlation was observed between *HvSDG5* and *HvSDG14*, and in *HvSDG19* and *HvPRMT1*. In *HDMs*, *HvJMJ11*, *HvJMJ8*,* HvJMJ6*, and *HvHDMA3* were positively correlated, while in *HAT*s, only one gene pair (*HvHAG1-HvHAG2*) was positively correlated. Among *HDAC*s, a positive correlation was observed for *HvHDA5*,* HvHDA14*,* HvHDA2*,* HvSRT2*, *HvHDA1*, and *HvHDT3.* Interestingly, in the DNA modifiers, the only correlation observed in the demethylases as *HvDME1* was negatively correlated to *HvDME3.* Among ATP-dependent chromatin remodelers, positive correlation was observed between *HvSnf2_2* with *HvChd1* and *HvEtl1*,* HvALC1 and HvMi_2* with *HvRad5_16_4* and *HvRis1_2*, and *HvLsh1* with *HvDRD1_5*, while negative correlation was observed between *HvDRD1_4* with *HvRad5_16_4* and *HvRis1_3*. In the control conditions, positive correlations were observed between *HvJMJ10* (*HDMs)* and *HvSDG8* (*HMTs*) and *HvJMJ8* (*HDMs*) with *HvSRT2* (*HDACs*). The negative correlations were observed between *HvIswi_*1 and *HvHDT1*, *HvSMARCAL1_1* and *HvSDG22*,* HvIswi_1* and *HvHAG2*, and *HvJMJ8* and *HvHDT1.*

### Leaf senescence leads to alteration in epigenetic regulation including changes in histone modification

**Fig. 8:**
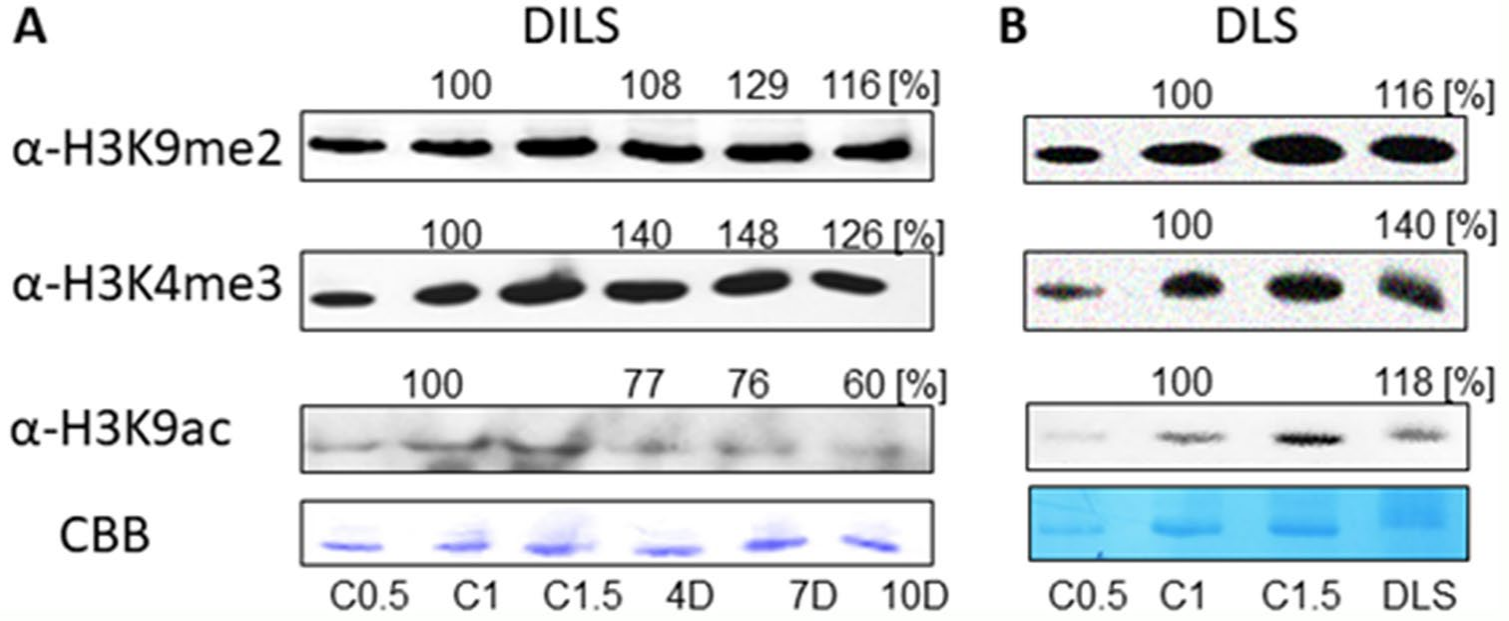
Changes of levels of heterochromatin (H3K9me2) and euchromatin (H3K4me3 and H3K9ac) histone marks during senescence. (**A**) Histone proteins extracted from primary leaves exposed to DILS, C- control plants (plants at day 0), D4- day 4 in the dark, D7- day 7 in the dark, D10- day 10 in the dark. (**B**) Histone proteins extracted from flag leaves during DLS, C- control plans (leaf 5 days before anthesis), DLS- 30th day post anthesis. Control (**C**) samples were loaded in three replications in every gel (in 0.5x, 1x, and 2x dilution) to prove the linear range of immunoreaction. Presented immunoblots are representative of at least three biological replications; 20–30 µg of chromatin protein was loaded on a gel. Coomassie brilliant blue staining (CBB) presents equal loading of gels

Our research has revealed considerable changes in histone modification levels during leaf senescence. From the range of antibodies tested on control material (Additional file 2: Fig. S9), the level of one heterochromatin (H3K9me2) and two euchromatin (H3K4me3 and H3K9ac) histone marks have been identified. Notably, an increase in H3 methylation levels during both DILS and DLS has been observed, while the acetylation level decreased exclusively during DILS (Fig. 8A, B, Additional file 2: Fig. S10-S15). These changes in histone modification levels seem to be specifically caused by the senescence, as in the control plants, the undisturbed development of leaves has not affected the histone modification status (Additional file 2: Fig. S16-S18).

## Discussion

### Genome-wide identification and characterisation of epigenetic regulatory genes in barley

Epigenetic regulatory mechanisms are crucial for controlling plant developmental processes and shaping phenotypic plasticity, including adaptive responses to environmental challenges. Epigenetic regulation is usually composed of histone modifications, chromatin remodelling, DNA methylation, and RNA interference (RNAi), which can be specific to particular internal or external conditions, and they can have phenotypic implications without directly altering the underlying DNA sequences [20, 21]. The understanding of the epigenetic regulatory machinery of plants has come mainly from genetic screens, most notably with the model plant *Arabidopsis thaliana*, which is highly amenable to genetic analyses. Among crops, primarily maize has contributed significantly to discovering epigenetic phenomena and epigenetic regulatory mechanisms [22]. In the past two decades, advancements in sequencing technologies and bioinformatics tools have been immense, and by utilising them, epigenetic regulatory genes have been identified in more plant species [23–25]. In this study, we identified 117 genes as the epigenetic regulators in barley at the whole genome scale, divided into three major classes: histone modifiers, DNA modifiers, and ATP-dependent chromatin remodelers. The different copy numbers of epigenetic regulatory genes in barley compared to other plants might be attributed to gene expansion and/or gene loss events [26]. The sequence alignment, protein domains, motif distribution, and phylogenetic analysis indicate that the epigenetic regulatory genes have shared the same evolutionary pattern and have similar functions to their plant homologs. The genome-to-genome synteny analysis between barley and four representative plant species exhibited a considerable syntenic correlation of epigenetic regulators within monocotyledons. However, very little orthologous correlation was found between the epigenetic regulatory genes in barley and *Arabidopsis*. Therefore, we speculated that the syntenic correlations between epigenetic regulators might be connected to the species’ evolutionary divergence.

The structural diversity of barley epigenetic regulatory genes was evaluated by assessing the distribution of exons and introns. Most were composed of multiple exons, and the number of exons/introns varied within the subfamilies. The variation in the number of introns is anticipated because the number and length of introns in genes vary depending on the organism and gene structure, and these differences may be related to intron function [27]. Moreover, the gain and loss of introns can change the structure of genes and play a vital role in the evolution of gene families [28]. Notably, histone modifiers contained single exon genes (*SEGs*) (e.g., H*vHDA10*,* HvSDG1*, and *HvSDG3*), given that *SEG* genes are prototypical of prokaryotes [29], their presence in multicellular eukaryotic genomes is intriguing. Furthermore, duplicated gene pairs were identified in the barley epigenetic genes, and the exon/intron gain/loss and divergence in exon/intron length were observed within the coding sequences of several of these genes. This could lead to generating functionally distinct paralogues [28]. However, these duplicated genes rarely vary in their biochemical activity but are limited to regulatory control variations [30]. The divergence time for the duplicated genes was estimated to be in the range of 15.3 (*HvSDG28/HvSDG20*) to 74.8 (*HvHAC2/HvHAC3*) MYA, suggesting a lineage-specific second duplication contemporaneous with grass species divergence (56–73 MYA) [31]. This also justifies the clustering of barley’s epigenetic genes with their homologs in the monocots. The duplicated gene pairs’ Ka/Ks values were less than one. This supports the possibility that genes may have evolved from intensive purifying selection pressure by natural selection during the evolutionary process [32].

We investigated the potential regulatory mechanism that controls the expression of epigenetic regulatory genes in barley by looking into *cis*-acting regulatory elements (CREs), transcription factor binding sites (TFBs), and CpG/CpNpG islands in the promoter regions. We identified several CREs taking part in multiple biological processes. We found many CAAT-box and TATA-box elements in the promoter regions of epigenetic regulatory genes. This is not surprising since those two types of elements represent the growth and development related CREs and take part in regulating the frequency of expression and initiating the transcription [33]. The other major CREs identified on the promoter regions of most of the genes were STRE, MYB, MYC elements, and ABRE elements, supporting their role in several pathways contributing to plant growth and development and in various stress responses [34, 35]. The G-box and G-Box, light-responsive elements, were mainly identified in DNA and histone modifiers, which have been reported in the regulation of chlorophyll biosynthesis in *Arabidopsis* [36] and also play essential roles in early senescence of rice flag leaf [37]. This suggests these elements might play a crucial role in gene expression during the DILS compared to DLS. Interestingly, these elements were not overrepresented in the ATP-dependent chromatin remodelers. However, several of their genes appeared to have differential expression during DILS. This indicates that the expression of ATP-dependent chromatin remodelers might be independent of G-box elements and have different regulatory mechanisms. The TFbs analysis in the promoter regions identified TCP, AP2/ERF TFbs, bZIP, MYB, GATA, and bHLH as the major TFbs, which have been well reported in concerned with plant growth/development and biotic as well as abiotic stress responses across various plant species [38]. Interestingly, WRKY TFbs were not found in the promoter regions of *HvHAC2* (*HAT*s), *HvHDA1*, and *HvHDA13* (*HDAC*s) in histone modifiers, and *HvEtl1*, *HvChd1*, and *HvATRX* in the ATP-dependent chromatin remodelers. WRKY TFs are important in regulating transcriptional reprogramming associated with plant growth and development and biotic and abiotic stress management [39]. Their absence in these genes suggests that they might have evolved a different mechanism of gene regulation, which might be of interest to future studies. The CpG/CpNpG islands in promoter regions represent critical DNA methylation sites, resulting in gene silence [40, 41]. CpG/CpNpG islands were found in the promoter regions of several epigenetic regulatory genes, showing that DNA methylation may regulate their gene expression. Furthermore, tandem repeats (TRs) were also identified in the promoter regions, showing that in these genes, there is a higher probability of mutation accumulating during replication (known as polymerase slippage) [42], which might be of great interest in studying mutational analysis in the controlling gene expression.

MiRNAs have been reported to regulate the responses of plants to different stress conditions [43–45], and also leaf senescence [46, 47]. This study identified several miRNAs with target sites in CDS of epigenetic regulatory genes. For instance, hvu-miR6211 target site were identified in *HvHDT3* gene, and hvu-miR5053 target site in *HvALC1*gene. The expression of *HvHDT3* was high in both DILS and DLS conditions, while *HvALC1*gene was highly expressed only in DILS. Therefore, it implies that hvu-miR6211 might be important in regulating the overall senescence process. However, hvu-miR5053 is only DILS-specific. This is an exciting finding, and the relationship between these miRNAs/target genes might be explored further to understand the complex mechanism of leaf senescence regulation. Furthermore, in this study, the regulatory protein-protein interactions (PPIs) network for the barley epigenetic regulatory proteins indicated considerable interactive networks among these and several other proteins. This is not surprising since PPIs represent an essential aspect of plant systems biology [48], and imply that barley epigenetic regulators participate in protein complexes with essential roles in various regulatory processes, cellular functions, and signalling cascades.

Using the publicly available database, we further analysed the expression profiles of barley epigenetic regulatory genes in different abiotic stress conditions. The differential expression of many *HvERGs* suggests their crucial role in regulating and adapting to unfavourable climatic conditions. This is unsurprising as several studies have reported the cross-talk between plants’ abiotic stress response pathways and epigenetic regulatory pathways (reviewed in [49–51]). However, there is still a knowledge gap in mechanisms underlying the correlation among different epigenetic phenomena occurring under various abiotic stresses in plants, and in particular, barley epigenetic regulators have not been thoroughly investigated so far. Thus, our findings identify the potential candidate epigenetic regulators for future studies and open a new direction in barley crop research.

### Regulation of epigenetic regulatory genes and levels of histone modification during leaf senescence in barley

A multilevel regulatory network controls leaf senescence, both developmental and stress-induced, and the dynamics of cooperation among all signal pathways are conditioned by plant phenotypic plasticity under different conditions [52]. We demonstrated that epigenetic mechanisms constitute another regulatory layer affecting the activity of senescence-associated mechanisms. In contrast to the gradual alteration that develops when the process occurs naturally, induced senescence involves a quick change in the genes responsible for epigenetic regulation.

The PCA analysis on the first two components, which explained 55.6% variability, divided the samples into three main groups: Control (light), DILS, and DLS. These results prove that although both DILS and DLS are senescence, the process differs regarding epigenetic regulation. The control group was mainly differentiated by *HvSDG22*,* HvHAC2*,* HvDME2*, and *HvHAG2* genes. The DILS group was distinguished by mainly *HvLsh_1*,* HvMot1*,* HvHAG2*,* HvHDA12*,* HvHAC2*, *HvERCC6_2*,* HvDME3*,* HvSDG22*, and *HvSRT2*. The DLS group was defined by *HvHAC3*,* HvSHPRH*,* HvRis1_2*,* HvDRD1_4*,* HvSMARCAL1_1*,* HvSnf2_2*,* HvHDT3*,* HvSDG8*,* HvJMJ11*,* HvALC1*,* HvSnf2_1*,* HvSDG27*, and *HvSnf2_3*.

The complex expression pattern highlights the complexity of chromatin remodelling in response to different senescence cues and the specific role of various chromatin remodelers in the senescence process. The correlation analyses indicate more significant trends among all epigenetic regulators in DILS. One of them is a mutually correlated group where a positive correlation is observed between the genes *HvDME2*,* HvSDG5*,* HvJMJ5*,* HvALC1*,* HvRad5_16_3*,* HvRis1_3*,* HvHAG1*,* HvMi2_1*, and a mutual negative correlation of these genes with *HvDRD1_4*,* HvHDA2*,* HvHvSRT2*.

These mutual correlations indicate a trend, illustrating the complexity of DILS at various epigenetic control levels and raising questions about possible interactions that require further investigation. The data presented here suggest that very few genes are exclusively DLS-regulated; these are *HvDRD1_4*, *HvSHPRH*, *HvDRM5*, and *HvHAC3*. Then, in this type of senescence, the regulations are mainly from the level of ATP-dependent chromatin remodelling. Meanwhile, induced senescence includes the sudden change in epigenetic regulator genes, which contrasts with the subtle shift that occurs when the process occurs naturally.

DNA methylation is the least involved in DILS. Rather, it is associated with increased demethylation activity. During DILS, there is an increase in the expression of DNA dimethyltransferases *HvDME1-2* and a decrease in the expression of methyltransferases *HvDRM5*,* HvMET* and *HvDRM4*.

The latest study conducted by Trejo-Arellano [53] presented significant downregulation of methylation pathway elements responsible for maintaining the integrity of the chromatin during dark-induced senescence, e.g., RdDM (RNA-directed DNA methylation) and DDM1/CMT2 (nucleosome remodelers: decreased DNA methylation 1/CHH methyltransferase) correlated with CHH methylation and heterochromatin at chromocenter decondensation. However, only local changes in methylome were present. A similar observation was made earlier in developmental senescence [54]. *DRD1*, whose decreased expression we observe in DILS, associated with darkness-induced senescence [55], has been identified as a synergistic component regulating DNA methylation with DDM1 in the RdDM-independent pathway [56].

During DILS, histone modifiers have differential regulation, with several histone lysine methyltransferases, demethylases, acetyltransferases, and deacetylases being upregulated while others are downregulated. The expression of the histone deacetylases *HvHDT1* and *HvHDA12* mainly shows upregulation during DILS, contrary to downregulation in DLS.

This may also be seen in the levels of histone modifications. H3K4me3 and H3K9me2 were increasing during both DILS and DLS progression. The level of H3K9ac dropped during DILS but not during DLS. In the control (light) conditions, histone modifications remained at the same level. This indicates the role of these modifications during the senescence process of the leaf. The diversity of histone modification types determines the complexity of their functions, just as their kind depends on the range of activity [57]. Histone modifications have already been shown as epigenetic marks involved in gene regulation in senescence. Both euchromatin marks H3K9ac and H3K4me3 were identified on a large number of SAGs and transcription factors upregulated during developmental senescence in leaves, where they appear at the high level in the early (H3K9ac) and the late stages (H3K4me3) of the senescence [58–60]. Similarly, the level of heterochromatic H3K9me2 was shown to be reduced around senescence-regulating genes, as well as global changes in H3K9me2 distribution in nuclei during barley leaf senescence were observed [54].

Moreover, on day 10 of DILS, numerous groups of genes belonging to chromatin remodelers, namely *HvIswi_2*, *HvChd1*, *HvRad54*, *HvDRD1_6*, *HvRad5_16_1* and *2*, *HvRis1_1*, and *HvERCC6_3*, become more expressed. DILS in barley occurs in two phases. The first phase is more strongly emphasised by the cessation of photosynthesis, loss of chlorophyll, and disintegration of chloroplasts. Despite the advanced state of macroautophagy in this phase, the degradation processes are reversible. The reversal of the DILS program involves regaining photosynthesis and increasing chlorophyll content, and it takes place irrespectively of the activation of ATG genes. The second, terminal phase, occurring beyond day 7 of darkness, is characterised by the irreversibility of senescence and its progression into PCD, exemplified by the involvement of both autophagy and PCD pathways, and involves disruption of the nucleus, mitochondria, chromatin condensation accompanied with nDNA fragmentation, shrinking of the protoplast, tonoplast interruption, and disintegration of the cell membrane [12]. Thus, ATP-dependent chromatin remodelling may condition metabolic reprogramming from senescence to PCD.

## Conclusions

The research presented here provides the first insight into the complete epigenetic machinery of barley and its contribution to the physiological process.

The provided study identifies and characterises epigenetic regulatory genes in barley at a whole-genome scale. It establishes insights into their genomic and structural organisation, regulatory framework, phylogenetic and evolutionary relationships, protein-protein interactions, and expression profiles under abiotic stresses, focusing on induced leaf senescence. This study is the first to analyse the barley epigenetic regulatory gene families systematically and comprehensively. The results present novel findings and offer valuable information on Gramineae crop development, stress physiology, and the prospects for genetic improvement programs related to epigenetics.

The findings suggest that epigenetic regulatory mechanisms are crucial for controlling plant development and facilitating adaptive responses to environmental stresses in barley. The correlations between specific genes in DILS highlight a complex epigenetic network responding to environmental cues. Histone modifications are also significant, with varying levels observed during senescence progression, highlighting their role in regulating chromatin dynamics.

From an evolutionary perspective, epigenetic regulator genes share similar patterns and functions with their counterparts in other plant species, especially other monocots. The structural diversity within these genes, particularly the presence of single exon genes and the variation in exon/intron counts, underscores their evolutionary adaptability and role in gene family evolution. Regulatory mechanisms controlling gene expression in barley involve multiple *cis*-acting regulatory elements and transcription factor binding sites, assisting in growth, development, and stress response pathways. miRNAs play a role in regulating barley epigenetic regulatory genes, with specific miRNAs targeting genes like *HvHDT3* and *HvALC1*, indicating their involvement in the senescence process. Protein-protein interaction networks suggest that barley epigenetic regulatory proteins participate in essential regulatory processes, cellular functions, and signalling cascades.

The study also supports an association between barley leaf senescence, specifically induced senescence, and the significant reorganisation of epigenetic regulation, gene transcriptomic, and histone modification levels. It emphasises the role of epigenetic mechanisms in regulating senescence-inducing signals, which environmental factors and the developmental program can influence. Induced senescence is a reversible process [12], and a strong capacity for survival characterises the specific metabolic strategies employed in response to darkening treatment [12, 61]. The level of control over senescence is achieved through various interdependent and mutually influencing epigenetic mechanisms. These mechanisms may enable a rapid response to signals and stimuli, allowing for the immediate adjustment of protein group expression and in induced senescence, ranging from dozens to thousands [7, 12], in response to fast-changing environmental conditions. The barley crop model of DILS, where the point of no return was defined, reveals differences in epigenetic regulatory gene expression compared to DLS. These results indicate that dark-induced leaf senescence, in the context of epigenetic regulation, is distinct from developmental senescence. Epigenetic regulation may act as a molecular switch between cell viability and cell death, operating according to the simple ‘live or die’ principle.

Mutual correlations in this work indicate a trend, illustrating the complexity of DILS at various epigenetic control levels and raising questions about possible interactions that require further investigation. However, the findings suggest that epigenetic regulatory mechanisms are crucial for controlling plant development and facilitating adaptive responses to environmental stresses in barley. Consequently, these findings could prove valuable in developing novel breeding strategies centred around epigenetic diversity (referred to as epi-breeding). Epigenetic variations within crops might serve as an additional and timely asset for enhancing crop breeding. Additionally, environmental stressors could serve as sources of epigenetic variation for improving physiological traits in crops, much like what has been done to strengthen rice’s drought and salt tolerance [62]. Utilising molecular epigenetic markers, akin to genetic markers, could aid in selection trials. Therefore, there is a need to investigate further the role of epigenetic regulators in crops during stress-induced senescence and explore their underlying molecular mechanisms. Understanding how epigenetic regulators and their regulatory networks function in this process within crops could be a valuable tool in advancing sustainable agriculture.

## Methods

### Plant material and growth conditions

Barley plants (*Hordeum vulgare* cv. Golden Promise) were grown as described previously [63]. For DILS experiments, 7-day-old seedlings were transferred to dark while the control (light) plants were grown continuously under the photoperiod. The primary leaves of seedlings were collected at days 0, 4, 7, and 10 of treatment. Prof. Per L. Gregersen (Arhus University, Denmark) kindly provided the leaf samples for DLS. The DLS leaves (senescent flag leaves − 30 days post anthesis, control leaves − 5 days before anthesis) and DILS leaves were snap-frozen in liquid nitrogen directly after collection and stored at -80 °C until further use.

### Identification and *in silico* characterisation of epigenetic regulatory genes and proteins in barley

To identify the epigenetic regulatory gene families in barley, known sequences of the histone acetyltransferase, histone deacetylase, histone methyltransferase, histone demethylase, DNA methyltransferase, DNA demethylase and ATP-dependent chromatin remodeler gene families of rice and *Arabidopsis* were used for a BLASTp search (E value, 1^− 10^) against the barley genome database hosted at Ensembl Plants (https://plants.ensembl.org/index.html*).* Barley protein sequences were analysed using Hidden Markov Model (HMM) for the presence of a typical HMM domain representing the corresponding protein class in an HMMER search (https://www.ebi.ac.uk/Tools/hmmer/*)* [64, 65]. Furthermore, these sequences were also cross-verified with the Inter-pro scan program hosted by a web tool (http://www.ebi.ac.uk/interpro/*)* [66]. The sub-cellular localisations were predicted by Plant-mPLoc webtool (http://www.csbio.sjtu.edu.cn/bioinf/plant-multi/*).* The MEME tool from the MEME suite 5.3.3 (http://meme-suite.org/tools/meme*)* was used to identify ten statistically significant motifs in the protein sequences based on “zero or one occurrence per sequence (zoops)” [67]. For gene structure analysis, the GFF3/GTF annotation file and genome assembly were extracted from the Ensembl Plants database (https://plants.ensembl.org/index.html*).* The gene structure, conserved motifs, and domains were visualised using the TBtools software [68].

### Evolutionary analysis of epigenetic regulators in barley

The protein sequences of reported epigenetic regulators of *A. thaliana*, *O. sativa*,* S. lycopersicum*, and *S.bicolor* were retrieved, and a phylogenetic tree was constructed using the Neighbor-Joining method with Poisson correction and 1000 bootstrap values using the MEGA-11 program [69]. The tree was visualised using the iTOLv6 program (https://itol.embl.de/*).* To analyse the synteny relationships of the orthologous genes among barley and other species, genome data and the gene annotation files of *A. thaliana*, *O. sativa*, *B. distachyon*, and *S. bicolor* were obtained from the Phytozome database (https://phytozome-next.jgi.doe.gov/*).* The syntenic analysis graphs were constructed by using the Dual Synteny Plotter function in TBtools software. Gene duplication events for barley epigenetic regulatory genes were analysed using the Multiple Collinearity Scan toolkit (MCScanX) with the default parameters and drawn by TBtools software. The number of synonymous (Ks) and non-synonymous (Ka) substitutions per site of duplicated gene pair were calculated by TBtools software. Based on a rate of 6.5 × 10^− 9^ substitutions per site per year, the divergence time (T) was calculated as T = Ks/(2 × 6.5 × 10^− 9^) × 10^− 6^ MYA for monocots [70].

### Promoter analysis, microRNA target site, and protein-protein interaction predictions

The Ensembl Plants database was used to obtain 1.5 kb sequences upstream of the translation start sites to analyse the promoter regions of epigenetic regulatory genes. The *cis*-acting regulatory elements (CREs) were identified using the PlantCARE database (http://bioinformatics.psb.ugent.be/webtools/plantcare/html/*)*. The PlantPAN 3.0 software (http://plantpan.itps.ncku.edu.tw/promoter.php*)* was used to identify transcription factor binding sites (TFbs) in the promoter regions, and the multiple promoter analysis program (http://plantpan2.itps.ncku.edu.tw/gene_group.php?#multipromoters*)* was used to identify the common TFbs. The prediction of CpG/CpNpG islands and tandem repeats (TRs) was made by the PlantPAN 3.0 web server (http://plantpan.itps.ncku.edu.tw/index.html*).* The coding sequences of barley epigenetic regulatory genes were analysed by the psRNATarget server (https://www.zhaolab.org/psRNATarget/*)* for miRNA target site prediction. The protein-protein interactions of epigenetic regulatory proteins were determined using the STRING web tool (https://string-db.org/) [71], and the network was generated using Cytoscape-3.9.0 software.

### Phenotyping of senescence progression

The Plant Screen high-throughput phenotyping system (Photon Systems Instruments, Czech Republic) was used to quantify the dynamic of plant senescence. The system allows non-invasive identification of stress symptoms from the early to the late stages by red–green–blue (RGB) morphometry, leaf pigment imaging, and chlorophyll fluorescence measurements. Intact barley plants were manually transferred to the imaging unit, where after 20 min of dark pre-incubation, the chlorophyll *a* fluorescence analyses were made using a Quenching protocol pre-designed by the manufacturer using 400 µmol photons m^− 2^ s^− 1^ actinic white light to evaluate PSII efficiency in a light-adapted state [72]. Leaf colour segmentation was used in RGB imaging to assess leaves’ loss of green pigment during senescence. Selection of hues belonging to three categories: ‘dark green, ‘light green,’ and ‘yellow’, made by the eye in processed RGB plant image and then clustered into ten k-means per category [72, 73].

### Gene expression analysis

The expression data of barley epigenetic regulatory genes under abiotic stress conditions were extracted from the barley expression database (BarleyExpDB, http://barleyexp.com/*)* as FPKM values. The expression patterns under heat, cold, dark/light, nitrogen, drought, and waterlogging stress conditions were selected and visualised by heat maps generated using the TBtools software. RNA-Seq data of barley developed in our lab (*Hordeum vulgare* cv. Golden Promise; BioProject: PRJNA962050; [74]) - were utilised for transcriptomic analysis of epigenetic regulatory genes in DILS and DLS. The differentially expressed genes (DEGs) were identified as described by Stolarska et al. [74]. Cleaned reads were mapped to the barley reference genome assembly using STAR aligner (version 2.7.10a), and raw counts of the mapped reads were quantified by FEATURECOUNT (version 2.0.3). The reads mapped to multiple loci were discarded. The R package ‘DESeq2’ was used to standardize the counts of each sample gene (use basemean value to estimate the expression), calculate the difference multiple, and use NB (negative binomial distribution test) to test the difference significance of the reads number. The differential protein coding genes were screened according to the difference multiple and difference significance test results. Finally, differentially expressed genes (DEGs) were identified based on Benjamini-Hochberg false discovery rate adjusted p-value of 0.05, and log2FC values were used to determine the up- or down-regulated transcripts.

### Statistical analysis

The transcriptomic gene expression data of epigenetic regulatory genes were further used for the statistical analyses using the R-language Version 4.3.1. The Principal Component Analysis (PCA) was performed using the PCA() function implemented in the “FactoMineR” package to estimate the number of components, and hierarchical clustering on the retained principal components (HCPC) was performed using the HCPC() function. The graphical outputs were visualised using the function fviz_cluster() of the “factoextra” package. Furthermore, associations among the differentially expressed epigenetic regulatory genes during DILS were sought, and their significance was tested using Pearson’s correlation tests. The correlation strength and significance (*P* < 0.05) were illustrated using the “corrplot” and “GGally” packages and visualised using the “ggplot2” package.

### Histone proteins extraction

Histone proteins were extracted from barley leaves, according to Sura et al. [75]. Frozen leaf samples were ground in liquid nitrogen and mixed with Honda buffer. Then, the homogenate was incubated on ice for 15 min, filtered through Miracloth, and centrifuged for 15 min at 2880 g at 4 °C. The pellet was resuspended gently in Honda buffer and centrifuged again. The procedure was repeated to obtain a white pellet of cell nuclei. The final wash of the cell nucleus pellet was performed in a buffer without spermine. The pellet was resuspended in 300 µl of cold TNE buffer, transferred to a 1.5 ml tube, and stored at -80 °C until further use.

### Western blot analysis

Histone protein extracts were solubilised with 4x Laemmli Buffer [76] and separated on 15% SDS-PAGE gels followed by electrotransfer onto PVDF membrane. The membranes were blocked in 3% bovine serum albumin (Sigma) and immunodetected with the ECL system (Bio-Rad) using the ChemiDoc™MP Imaging System (Bio-Rad). To detect histone posttranslational modifications, the following primary antibodies were used: H3K27me3 (1:1000; Abcam, Ab6002); H3K9me2 (1:2000; Abcam, Ab1220); H2K9me3 (1:1000; Abcam, ab8898); H3K4me3 (1:1000; Abcam, Ab8580); H3K36me3 (1:1000; Abcam, Ab9050); H3K14ac (1:1000; Milipore, 07-353); H3K9ac (1:500; Milipore, 07-352); H3K27me2/me3 (1: 1000; Abcam, Ab6147); H3K4me3 (1:1000; Milipore, 07-473); H3K27ac (Abcam, 1:1000, Ab4727), H3 (1:5000, Agrisera, AS10 710). The quantification of immunostained signals was performed using Gelix One software.

## Electronic supplementary material

Below is the link to the electronic supplementary material.

Supplementary Material 1

Supplementary Material 2

## Data Availability

The data that support the findings of this study is available at Ensembl Plants database (https://plants.ensembl.org/index.html) or can be obtained from the corresponding author upon reasonable request. The RNA-Seq data used in this study is available at NCBI-SRA database, BioProject: PRJNA962050.

## Abbreviations

CDS: Coding sequence
CREs: Cis-acting regulatory elements
DEG: Differentially Expressed Gene
DILS: Dark induced leaf senescence
DLS: Developmental leaf senescence
DNMT: DNA methyltransferase
DME: DNA demethylase
H3K4me3: Histone H3 at lysine 4 trimethylation
H3K9ac: Histone H3 at lysine 9 acetylation
H3K9me2: Histone H3 at lysine 9 dimethylation
HAT: Histone acetyltransferase
HDAC: Histone deacetylase
HDM: Histone demethylase
HMT: Histone methyltransferase
Hv: Hordeum vulgare
PCA: Principal Component Analysis
PPI: Protein-protein interaction
QY_max_: Maximum efficiency of primary photochemistry
RdDM: RNA-directed DNA methylation
RNAi: RNA interference
SAG: Senescence-associated gene
SEG: Single exon gene
TF: Transcription factor
TE: Transposable element
TFB: Transcription factor binding site
TR: Tandem repeat

## References

[1] Thomas H. Senescence, ageing and death of the whole plant. New Phytol. 2013;197(3):696–711. 23176101 10.1111/nph.12047

[2] Wojciechowska N, Sobieszczuk-Nowicka E, Bagniewska-Zadworna A. Plant organ senescence - regulation by manifold pathways. Plant Biol (Stuttg). 2018;20(2):167–81. 29178615 10.1111/plb.12672

[3] Paluch-Lubawa E, Stolarska E, Sobieszczuk-Nowicka E. Dark-Induced Barley Leaf Senescence - A Crop System for studying senescence and autophagy mechanisms. Front Plant Sci. 2021;12:635619. 33790925 10.3389/fpls.2021.635619 PMC8005711

[4] Christiansen MW, Gregersen PL. Members of the barley NAC transcription factor gene family show differential co-regulation with senescence-associated genes during senescence of flag leaves. J Exp Bot. 2014;65(14):4009–22. 24567495 10.1093/jxb/eru046 PMC4106437

[5] Sobieszczuk-Nowicka E, Zmienko A, Samelak-Czajka A, Łuczak M, Pietrowska-Borek M, Iorio R, et al. Dark-induced senescence of barley leaves involves activation of plastid transglutaminases. Amino Acids. 2015;47(4):825–38. 25583605 10.1007/s00726-014-1912-y PMC4361728

[6] Sobieszczuk-Nowicka E, Kubala S, Zmienko A, Małecka A, Legocka J. From Accumulation to Degradation: Reprogramming Polyamine Metabolism facilitates Dark-Induced Senescence in Barley Leaf cells. Front Plant Sci. 2016;6:1198. 26779231 10.3389/fpls.2015.01198 PMC4702279

[7] Law SR, Chrobok D, Juvany M, Delhomme N, Lindén P, Brouwer B, et al. Darkened leaves use different metabolic strategies for senescence and survival. Plant Physiol. 2018;177(1):132–50. 29523713 10.1104/pp.18.00062 PMC5933110

[8] Gregersen PL, Holm PB, Krupinska K. Leaf senescence and nutrient remobilisation in barley and wheat. Plant Biol (Stuttg). 2008;10(1):37–49. 18721310 10.1111/j.1438-8677.2008.00114.x

[9] Buchanan-Wollaston V, Earl S, Harrison E, Mathas E, Navabpour S, Page T, et al. The molecular analysis of leaf senescence–a genomics approach. Plant Biotechnol J. 2003;1(1):3–22. 17147676 10.1046/j.1467-7652.2003.00004.x

[10] Buchanan-Wollaston V, Page T, Harrison E, Breeze E, Pyung OL, Hong GN, et al. Comparative transcriptome analysis reveals significant differences in gene expression and signalling pathways between developmental and dark/starvation-induced senescence in *Arabidopsis*. Plant J. 2005;42(4):567–85. 15860015 10.1111/j.1365-313X.2005.02399.x

[11] Breeze E, Harrison E, McHattie S, Hughes L, Hickman R, Hill C, et al. High-resolution temporal profiling of transcripts during *Arabidopsis* Leaf Senescence reveals a distinct chronology of processes and regulation. Plant Cell. 2011;23(3):873–94. 21447789 10.1105/tpc.111.083345 PMC3082270

[12] Sobieszczuk-Nowicka E, Wrzesiński T, Bagniewska-Zadworna A, Kubala S, Rucińska-Sobkowiak R, Polcyn W, et al. Physio-genetic dissection of dark-induced leaf senescence and timing its reversal in barley. Plant Physiol. 2018;178(2):654–71. 30126868 10.1104/pp.18.00516 PMC6181038

[13] Li Z, Zhao T, Liu J, Li H, Liu B. Shade-induced leaf senescence in plants. Plants (Basel). 2023;12(2):123–35. 10.3390/plants12071550 PMC10097262 37050176

[14] Wehner GG, Balko CC, Enders MM, Humbeck KK, Ordon FF. Identification of genomic regions involved in tolerance to drought stress and drought stress-induced leaf senescence in juvenile barley. BMC Plant Biol. 2015;15(1):1–16. 25998066 10.1186/s12870-015-0524-3 PMC4440603

[15] Kopecká R, Kameniarová M, Černý M, Brzobohatý B, Novák J. Abiotic stress in crop production. Int J Mol Sci. 2023;24(7):6603. 37047573 10.3390/ijms24076603 PMC10095105

[16] Gepstein S, Glick BR. Strategies to ameliorate abiotic stress-induced plant senescence. Plant Mol Biol. 2013;82(6):623–33. 23595200 10.1007/s11103-013-0038-z

[17] Hollmann J, Gregersen PL, Krupinska K. Identification of predominant genes involved in regulation and execution of senescence-associated nitrogen remobilization in flag leaves of field grown barley. J Exp Bot. 2014;65(14):3963–73. 24700620 10.1093/jxb/eru094 PMC4106439

[18] Ostrowska-Mazurek A, Kasprzak P, Kubala S, Zaborowska M, Sobieszczuk-Nowicka E. Epigenetic landmarks of Leaf Senescence and Crop Improvement. Int J Mol Sci. 2020;21(14):5125. 32698545 10.3390/ijms21145125 PMC7404090

[19] Lloyd JPB, Lister R. Epigenome plasticity in plants. Nat Rev Genet. 2022;23(1):55–68. 34526697 10.1038/s41576-021-00407-y

[20] Eriksson MC, Szukala A, Tian B, Paun O. Current research frontiers in plant epigenetics: an introduction to a virtual issue. New Phytol. 2020;226(2):285–8. 32180259 10.1111/nph.16493 PMC7154677

[21] Gallusci P, Dai Z, Génard M, Gauffretau A, Leblanc-Fournier N, Richard-Molard C, et al. Epigenetics for plant improvement: current knowledge and modeling avenues. Trends Plant Sci. 2017;22(7):610–23. 28587758 10.1016/j.tplants.2017.04.009

[22] Pikaard CS, Scheid OM. Epigenetic regulation in plants. Cold Spring Harb Perspect Biol. 2014;6(12):a019315. 25452385 10.1101/cshperspect.a019315 PMC4292151

[23] Ahmad F, Huang X, Lan HX, Huma T, Bao YM, Huang J, et al. Comprehensive gene expression analysis of the DNA (cytosine-5) methyltransferase family in rice (*Oryza sativa* L). Genet Mol Res. 2014;13(3):5159–72. 25061741 10.4238/2014.July.7.9

[24] Hu Y, Chen X, Zhou C, He Z, Shen X. Genome-wide identification of chromatin regulators in Sorghum bicolor. 3 Biotech. 2022;12(5):117. 35547013 10.1007/s13205-022-03181-8 PMC9033926

[25] Liu X, Luo M, Zhang W, Zhao J, Zhang J, Wu K, et al. Histone acetyltransferases in rice (Oryza sativa L.): phylogenetic analysis, subcellular localization and expression. BMC Plant Biol. 2012;12(1):145. 22894565 10.1186/1471-2229-12-145 PMC3502346

[26] Aoyagi BY, Satake A. Analyses of gene copy number variation in diverse epigenetic regulatory gene families across plants: increased copy numbers of BRUSHY1/TONSOKU/MGOUN3 (BRU1/TSK/MGO3) and SILENCING DEFECTIVE 3 (SDE3) in long-lived trees. Plant Gene. 2022;32:100359.

[27] Grabherr MG, Haas BJ, Yassour M, Levin JZ, Thompson DA, Amit I, et al. Full-length transcriptome assembly from RNA-Seq data without a reference genome. Nat Biotechnol. 2011;29(7):644–52. 21572440 10.1038/nbt.1883 PMC3571712

[28] Xu G, Guo C, Shan H, Kong H. Divergence of duplicate genes in exon-intron structure. Proc Natl Acad Sci U S A. 2012;109(4):1187–92. 22232673 10.1073/pnas.1109047109 PMC3268293

[29] Sakharkar MK, Chow VT, Chaturvedi I, Mathura VS, Shapshak P, Kangueane P. A report on single exon genes (SEG) in eukaryotes. Front Biosci. 2004;9:3262–7. 15353355 10.2741/1478

[30] Wapinski I, Pfeffer A, Friedman N, Regev A. Automatic genome-wide reconstruction of phylogenetic gene trees. Bioinformatics. 2007;23(13). 10.1093/bioinformatics/btm193 17646342

[31] Gaut BS. Evolutionary dynamics of grass genomes. New Phytol. 2002;154(1):15–28. 10.1046/j.1469-8137.2002.00352.x

[32] Hurst LD. The Ka/Ks ratio: diagnosing the form of sequence evolution. Trends Genet. 2002;18(9):486–7. 12175810 10.1016/S0168-9525(02)02722-1

[33] Laloum T, De Mita S, Gamas P, Baudin M, Niebel A. CCAAT-box binding transcription factors in plants: Y so many? Trends Plant Sci. 2013;18(2):157–66. 22939172 10.1016/j.tplants.2012.07.004

[34] Marand AP, Eveland AL, Kaufmann K, Springer NM. cis-Regulatory elements in plant development, adaptation, and evolution. Annu Rev Plant Biol. 2023;74:111–37. 36608347 10.1146/annurev-arplant-070122-030236 PMC9881396

[35] Nakashima K, Yamaguchi-Shinozaki K. ABA signaling in stress-response and seed development. Plant Cell Rep. 2013;32(7):959–70. 23535869 10.1007/s00299-013-1418-1

[36] Kobayashi K, Baba S, Obayashi T, Sato M, Toyooka K, Keränen M, et al. Regulation of root greening by light and auxin/cytokinin signaling in *Arabidopsis*. Plant Cell. 2012;24(3):1081–95. 22415275 10.1105/tpc.111.092254 PMC3336121

[37] Liu L, Xu W, Hu X, Liu H, Lin Y. W-box and G-box elements play important roles in early senescence of rice flag leaf. Sci Rep. 2016;6:20881. 26864250 10.1038/srep20881 PMC4749992

[38] Das A, Pramanik K, Sharma R, Gantait S, Banerjee J. In-silico study of biotic and abiotic stress-related transcription factor binding sites in the promoter regions of rice germin-like protein genes. PLoS ONE. 2019;14(2). 10.1371/journal.pone.0211887 PMC6375593 30763346

[39] Khoso MA, Hussain A, Ritonga FN, Ali Q, Channa MM, Alshegaihi RM, et al. WRKY transcription factors (TFs): molecular switches to regulate drought, temperature, and salinity stresses in plants. Front Plant Sci. 2022;13:839139. 10.3389/fpls.2022.1039329 PMC9679293 36426143

[40] Cao X, Jacobsen SE. Locus-specific control of asymmetric and CpNpG methylation by the DRM and CMT3 methyltransferase genes. Proc Natl Acad Sci U S A. 2002;99(Suppl 4):16491–8. 12151602 10.1073/pnas.162371599 PMC139913

[41] Zemach A, Grafi G. Methyl-CpG-binding domain proteins in plants: interpreters of DNA methylation. Trends Plant Sci. 2007;12(2):80–5. 17208509 10.1016/j.tplants.2006.12.004

[42] Ahmar S, Gruszka D. In-silico study of brassinosteroid signaling genes in rice provides insight into mechanisms which regulate their expression. Front Genet. 2022;13:929612. 10.3389/fgene.2022.953458 PMC9299959 35873468

[43] Kantar M, Unver T, Budak H. Regulation of barley miRNAs upon dehydration stress correlated with target gene expression. Funct Integr Genomics. 2010;10(4):493–507. 20676715 10.1007/s10142-010-0181-4

[44] Sunkar R, Li YF, Jagadeeswaran G. Functions of microRNAs in plant stress responses. Trends Plant Sci. 2012;17(4):196–203. 22365280 10.1016/j.tplants.2012.01.010

[45] Wu L, Yu J, Shen Q, Huang L, Wu D, Zhang G. Identification of microRNAs in response to aluminum stress in the roots of tibetan wild barley and cultivated barley. BMC Genomics. 2018;19(1). 10.1186/s12864-018-4953-x PMC6069884 30064381

[46] Huo X, Wang C, Teng Y, Liu X. Identification of miRNAs associated with dark-induced senescence in *Arabidopsis*. BMC Plant Biol. 2015;15(1). 10.1186/s12870-015-0656-5 PMC4632659 26530097

[47] Thatcher SR, Burd S, Wright C, Lers A, Green PJ. Differential expression of miRNAs and their target genes in senescing leaves and siliques: insights from deep sequencing of small RNAs and cleaved target RNAs. Plant Cell Environ. 2015;38(1):188–200. 24965556 10.1111/pce.12393 PMC4304344

[48] Struk S, Jacobs A, Sánchez Martín-Fontecha E, Gevaert K, Cubas P, Goormachtig S. Exploring the protein-protein interaction landscape in plants. Plant Cell Environ. 2019;42(2):387–409. 30156707 10.1111/pce.13433

[49] Chang YN, Zhu C, Jiang J, Zhang H, Zhu JK, Duan CG. Epigenetic regulation in plant abiotic stress responses. J Integr Plant Biol. 2020;62(5):563–80. 31872527 10.1111/jipb.12901

[50] Chinnusamy V, Zhu JK. Epigenetic regulation of stress responses in plants. Curr Opin Plant Biol. 2009;12(2):133–9. 19179104 10.1016/j.pbi.2008.12.006 PMC3139470

[51] Rehman M, Tanti B. Understanding epigenetic modifications in response to abiotic stresses in plants. Biocatal Agric Biotechnol. 2020;27:101673. 10.1016/j.bcab.2020.101673

[52] Miryeganeh M, Saze H. Epigenetic inheritance and plant evolution. Popul Ecol. 2020;62(1):17–27. 10.1002/1438-390X.12018

[53] Trejo-Arellano MS, Mehdi S, de Jonge J, Tomastíková ED, Köhler C, Hennig L. Dark-induced senescence causes localized changes in DNA methylation. Plant Physiol. 2020;182(2):949–61. 31792150 10.1104/pp.19.01154 PMC6997673

[54] Ay N, Irmler K, Fischer A, Uhlemann R, Reuter G, Humbeck K. Epigenetic programming via histone methylation at WRKY53 controls leaf senescence in *Arabidopsis thaliana*. Plant J. 2009;58(2):333–46. 19143996 10.1111/j.0960-7412.2009.03782.x

[55] Cho EJ, Choi SH, Kim JH, Kim JE, Lee MH, Chung BY et al. A mutation in plant-specific SWI2/SNF2-Like chromatin-remodeling proteins, DRD1 and DDM1, Delays Leaf Senescence in *Arabidopsis thaliana*. PLoS ONE. 2016;11(1). 10.1371/journal.pone.0146826 PMC4709239 26752684

[56] Zemach A, Kim MY, Hsieh PH, Coleman-Derr D, Eshed-Williams L, Thao K, et al. The *Arabidopsis* nucleosome remodeler DDM1 allows DNA methyltransferases to access H1-containing heterochromatin. Cell. 2013;153(1):193–205. 23540698 10.1016/j.cell.2013.02.033 PMC4035305

[57] Liu C, Lu F, Cui X, Cao X. Histone methylation in higher plants. Annu Rev Plant Biol. 2010;61:395–420. 20192747 10.1146/annurev.arplant.043008.091939

[58] Brusslan JA, Rus Alvarez-Canterbury AM, Nair NU, Rice JC, Hitchler MJ, Pellegrini M. Genome-wide evaluation of histone methylation changes associated with leaf senescence in *Arabidopsis*. PLoS ONE. 2012;7(3). 10.1371/journal.pone.0033151 PMC3299739 22427974

[59] Brusslan JA, Bonora G, Rus-Canterbury AM, Tariq F, Jaroszewicz A, Pellegrini M. A genome-wide chronological study of gene expression and two histone modifications, H3K4me3 and H3K9ac, during developmental leaf senescence. Plant Physiol. 2015;168(4):1246–61. 25802367 10.1104/pp.114.252999 PMC4528724

[60] Yan H, Liu Y, Zhang K, Song J, Xu W, Su Z. Chromatin state-based analysis of epigenetic H3K4me3 marks of *Arabidopsis* in response to dark stress. Front Genet. 2019;10:306. 31001332 10.3389/fgene.2019.00306 PMC6456666

[61] Kleber-Janke T, Krupinska K. Isolation of cDNA clones for genes showing enhanced expression in barley leaves during dark-induced senescence as well as during senescence under field conditions. Planta. 1997;203(3):332–40. 9431680 10.1007/s004250050199

[62] Garg R, Narayana Chevala V, Shankar R, Jain M. Divergent DNA methylation patterns associated with gene expression in rice cultivars with contrasting drought and salinity stress response. Sci Rep. 2015;5:14922. 26449881 10.1038/srep14922 PMC4598828

[63] Tanwar UK, Stolarska E, Paluch-Lubawa E, Mattoo AK, Arasimowicz-Jelonek M, Sobieszczuk-Nowicka E. Unraveling the genetics of polyamine metabolism in barley for senescence-related crop improvement. Int J Biol Macromol. 2022;221:585–603. 36075308 10.1016/j.ijbiomac.2022.09.006

[64] Finn RD, Clements J, Eddy SR. HMMER web server: interactive sequence similarity searching. Nucleic Acids Res. 2011;39. 10.1093/nar/gkr367 PMC3125773 21593126

[65] Upadhyay RK, Mattoo AK. Genome-wide identification of tomato (*Solanum lycopersicum* L.) lipoxygenases coupled with expression profiles during plant development and in response to methyl-jasmonate and wounding. J Plant Physiol. 2018;231:318–28. 30368230 10.1016/j.jplph.2018.10.001

[66] Finn RD, Attwood TK, Babbitt PC, Bateman A, Bork P, Bridge AJ, et al. InterPro in 2017-beyond protein family and domain annotations. Nucleic Acids Res. 2017;45:D1. 27899635 10.1093/nar/gkw1107 PMC5210578

[67] Bailey TL, Boden M, Buske FA, Frith M, Grant CE, Clementi L, Ren J, Li WW, Noble WS. MEME SUITE: tools for motif discovery and searching. Nucleic Acids Res. 2009;37. 10.1093/nar/gkp335 PMC2703892 19458158

[68] Chen C, Chen H, Zhang Y, Thomas HR, Frank MH, He Y, Xia R. TBtools: an integrative Toolkit developed for interactive analyses of big Biological Data. Mol Plant. 2020;13(8):1194–202. 32585190 10.1016/j.molp.2020.06.009

[69] Tamura K, Stecher G, Kumar S. MEGA11: Molecular Evolutionary Genetics Analysis Version 11. Mol Biol Evol. 2021;38(7):3022–7. 33892491 10.1093/molbev/msab120 PMC8233496

[70] Ju L, Deng G, Liang J, Zhang H, Li Q, Pan Z, Yu M, Long H. Structural organization and functional divergence of high isoelectric point α-amylase genes in bread wheat (*Triticum aestivum* L.) and barley (*Hordeum vulgare* L). BMC Genet. 2019;20(1):25. 30845909 10.1186/s12863-019-0732-1 PMC6404323

[71] Szklarczyk D, Gable AL, Nastou KC, Lyon D, Kirsch R, Pyysalo S, et al. The STRING database in 2021: customizable protein-protein networks, and functional characterization of user-uploaded gene/measurement sets. Nucleic Acids Res. 2021;49:D1. 33237311 10.1093/nar/gkab835 PMC7779004

[72] Arasimowicz-Jelonek M, Jagodzik P, Płóciennik A, Sobieszczuk-Nowicka E, Mattoo A, Polcyn W, Floryszak-Wieczorek J. Dynamics of nitration during dark-induced leaf senescence in *Arabidopsis* reveals proteins modified by tryptophan nitration. J Exp Bot. 2022;73(19):6853–75. 35981877 10.1093/jxb/erac341

[73] Hassan MR, Ema RR, Islam T. Color Image Segmentation using Automated K-Means clustering with RGB and HSV Color spaces. GJCST. 2017;17(F2):33–41.

[74] Stolarska E, Tanwar UK, Guan Y, Grabsztunowicz M, Arasimowicz-Jelonek M, Phanstiel O, et al. Genetic portrait of polyamine transporters in barley: insights in the regulation of leaf senescence. Front Plant Sci. 2023;14:1133. 10.3389/fpls.2023.1194737 PMC10272464 37332717

[75] Sura W, Kabza M, Karlowski WM, Bieluszewski T, Kus-Slowinska M, Pawełoszek Ł, et al. Dual role of the histone variant H2A.Z in transcriptional regulation of stress-response genes. Plant Cell. 2017;29(4):791–807. 28258158 10.1105/tpc.16.00573 PMC5435421

[76] Laemmli UK. Cleavage of structural proteins during the assembly of the head of bacteriophage T4. Nature. 1970;227(5259):680–5. 5432063 10.1038/227680a0

