# Supplementary material for: Unveiling the role of epigenetics in leaf senescence: a comparative study to identify different epigenetic regulations of senescence types in barley leaves
Source: BMC Plant Biol. 2024 Sep 14;24:863. doi: 10.1186/s12870-024-05573-9 (PMC11401419; doi:10.1186/s12870-024-05573-9)
Supplement: Supplementary file 2 — Supplementary Material 2 [file 12870_2024_5573_MOESM2_ESM.docx]

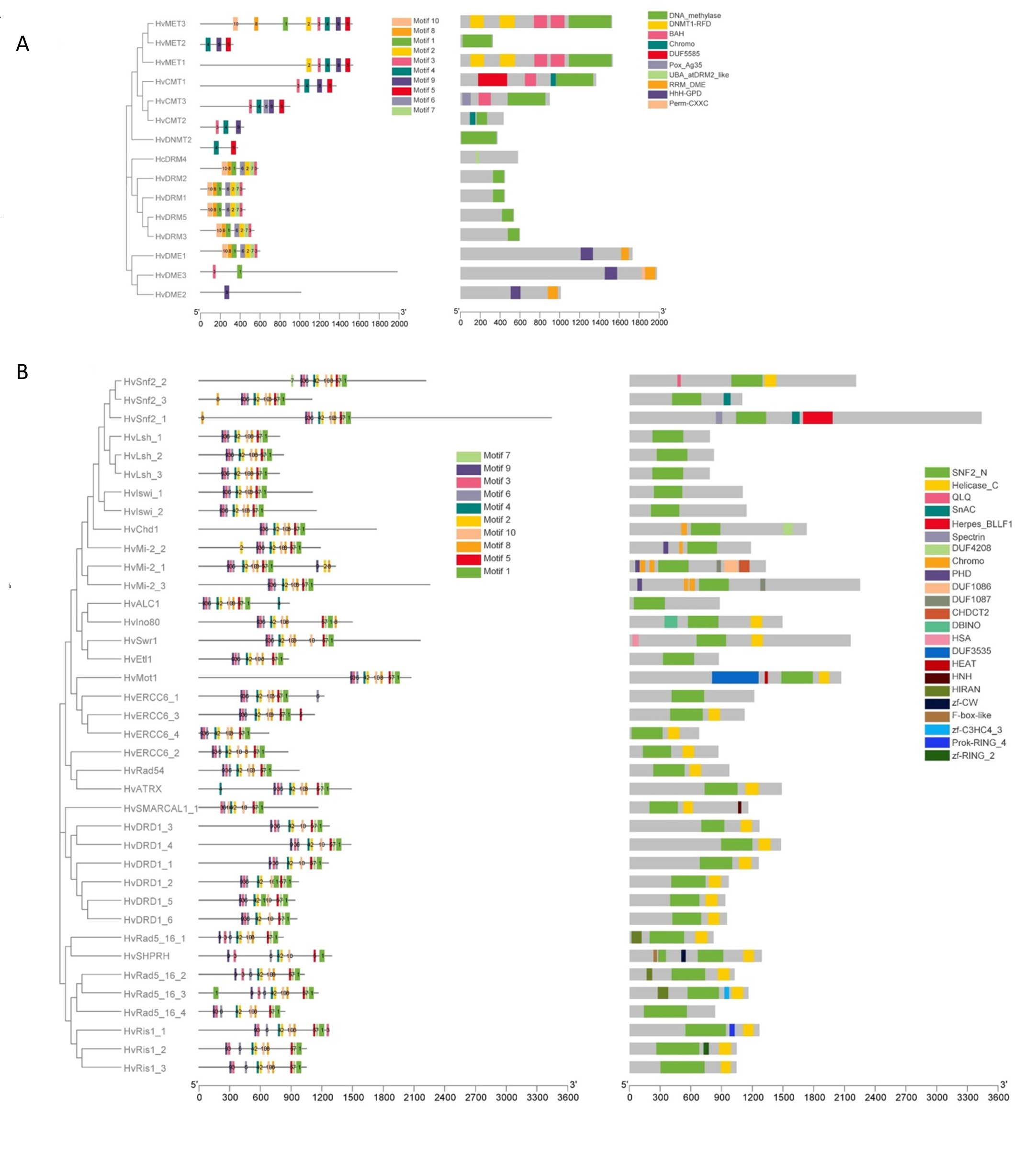


**Fig. S1.** Distributions of the conserved motifs and domains in **A** DNA modifying proteins and in **B** ATP-dependent chromatin remodelers proteins from barley.


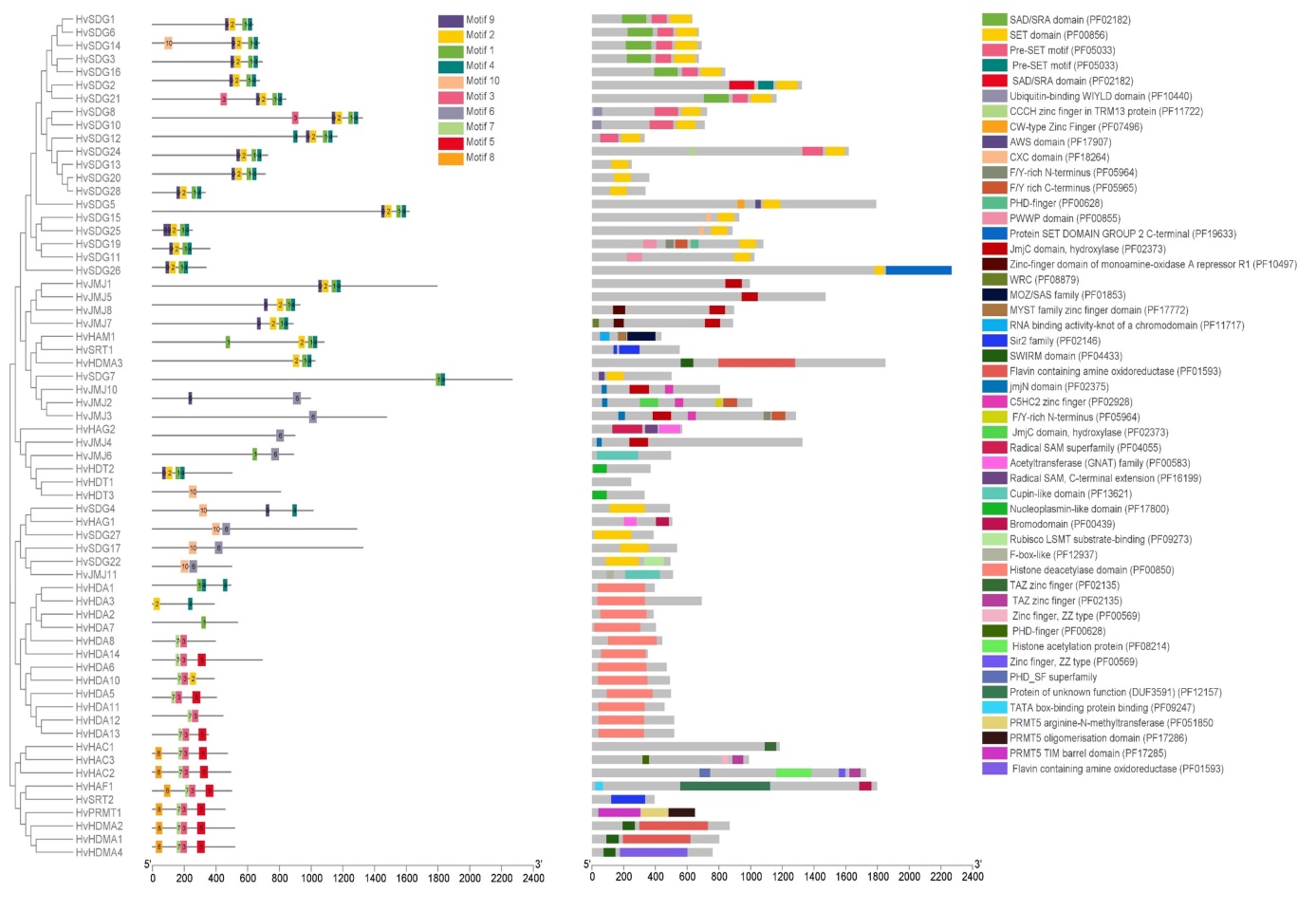


**Fig. S2.** Distributions of the conserved motifs and domains in histone modifying proteins from barley.

**
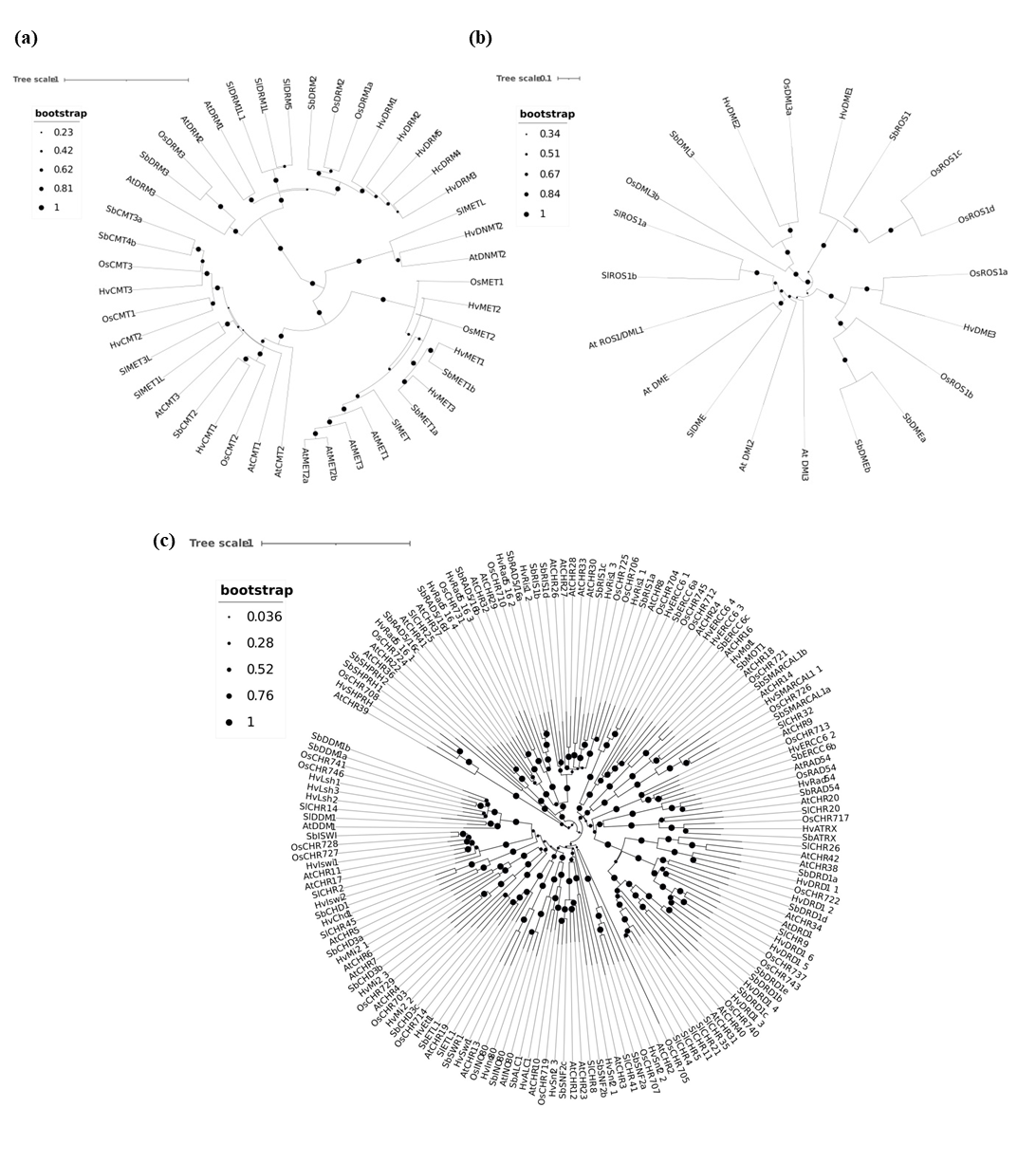
**

**Fig. S3.** Phylogenetic relationship of DNA methyltransferase (a), DNA demethylase (b) and ATP-dependent chromatin remodeler (c) protein sequences from *H. vulgare* (*Hv*) and other plant species: *A. thaliana* (*At*), *O. sativa* (*Os*), *S. bicolor* (*Sb*), and *S. lycopersicum* (*Sl*).

**
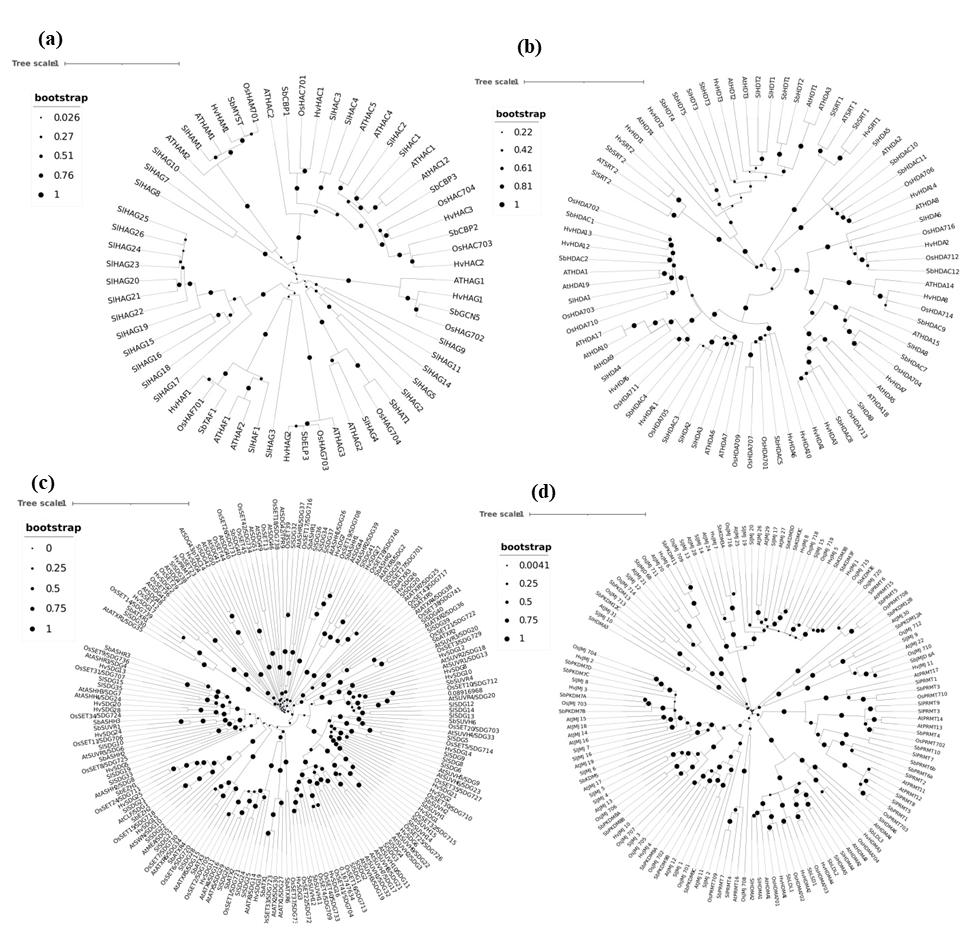
**

**Fig. S4.** Phylogenetic relationship of histone acetyltransferase (a), histone deacetylase (b), histone methyltransferase (c) and histone demethylase (d) protein sequences from *H. vulgare* (*Hv*) and other plant species: *A. thaliana* (*At*), *O. sativa* (*Os*), *S. bicolor* (*Sb*), and *S. lycopersicum* (*Sl*).

C


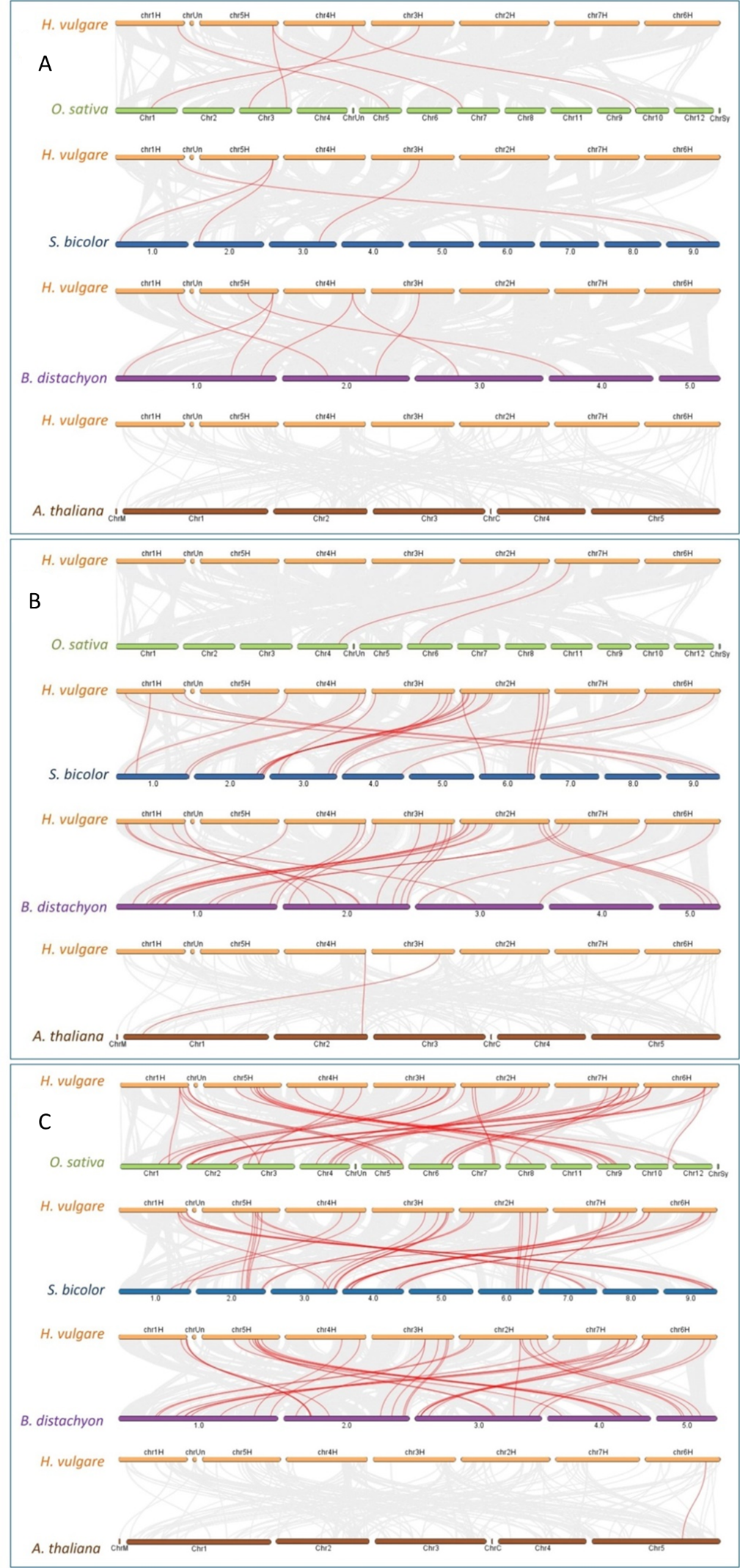


**Fig. S5.** Synteny analysis of epigenetic regulatory genes in *H. vulgare* with other plant species: *A. thaliana, B. distachyon, O. sativa*, and *S. bicolor*. **A** DNA modifiers, **B** ATP-dependent chromatin remodelers, **C** histone modifiers**.**


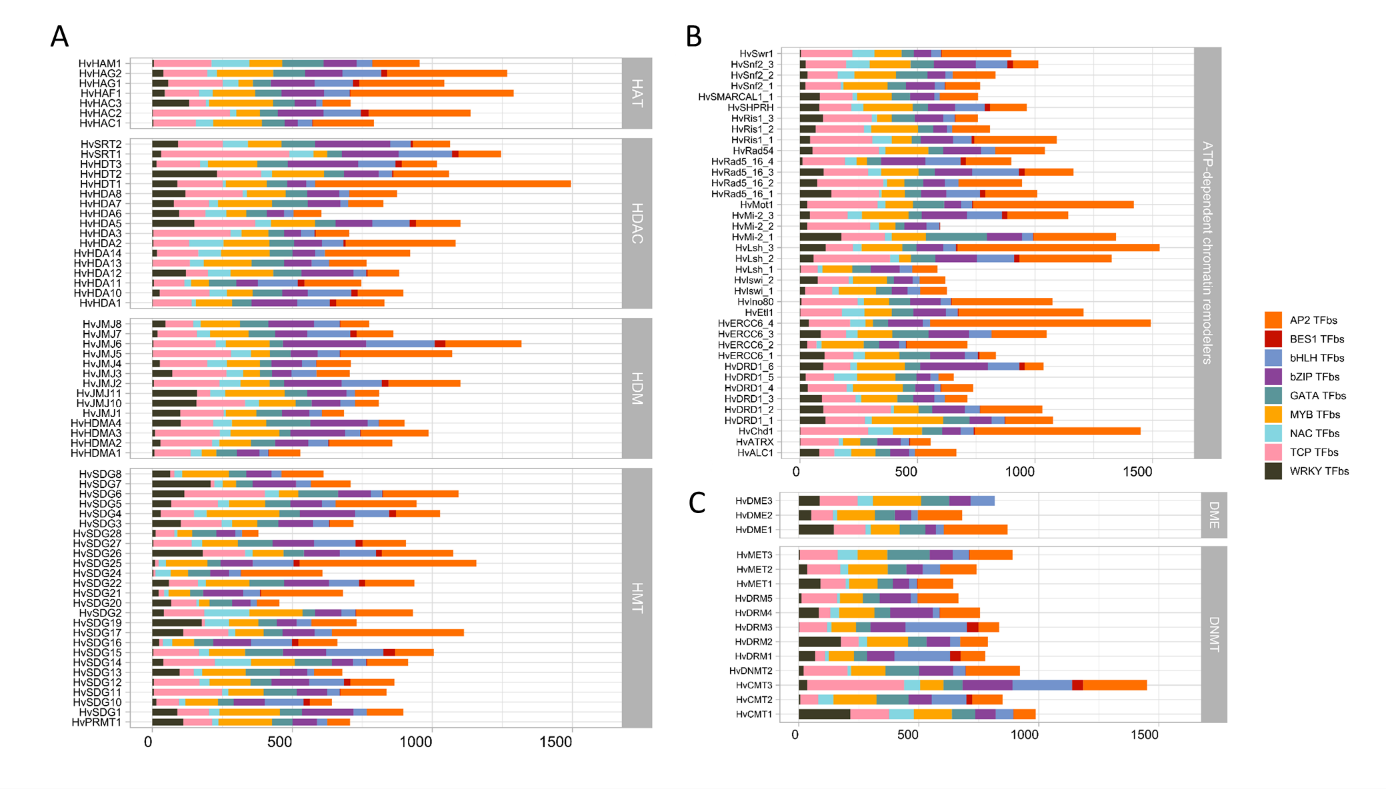


**Fig. S6**. Transcription factor binding sites in the promoter regions of barley epigenetic regulatory genes. **A.** Histone modifiers, **B.** ATP-dependent chromatin remodelers, and **C.** DNA modifiers.


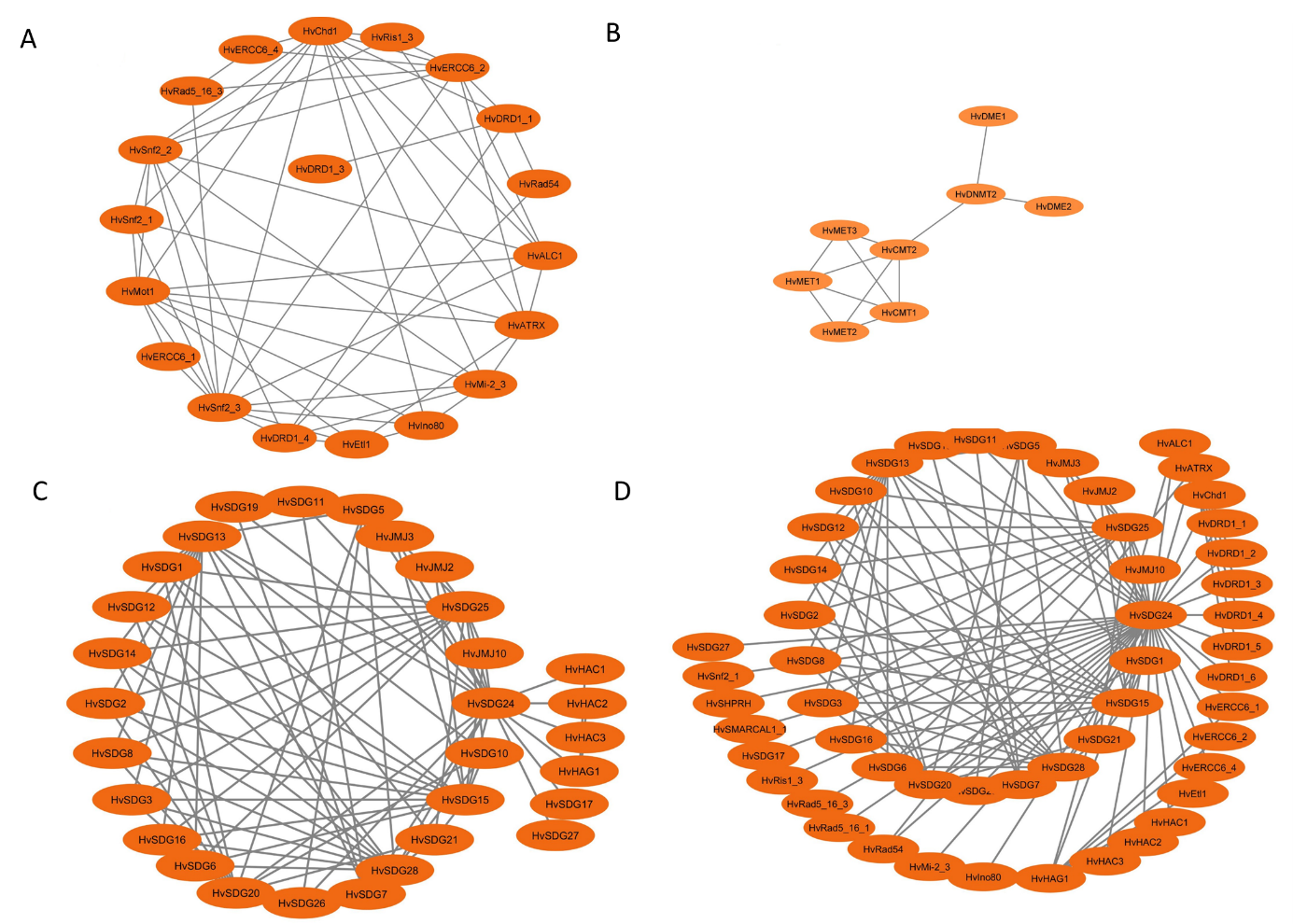


B

**Fig. S7.** Protein-protein interactions network of epigenetic regulatory genes in barley. **A** ATP-dependent chromatin remodelers, **B** DNA modifiers, **C** histone modifiers, **D** all epigenetic regulators.


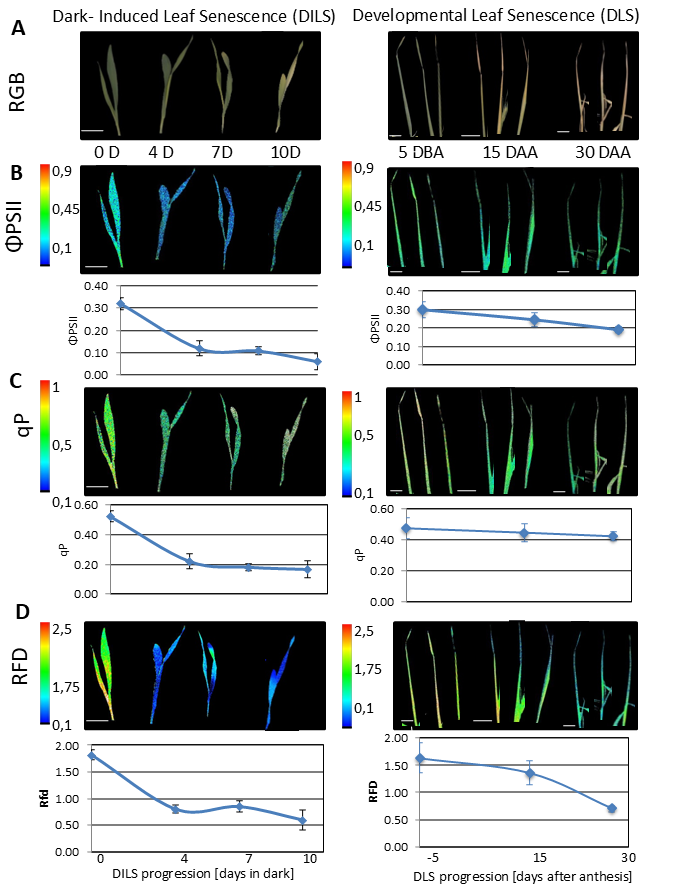


**Fig S8.** Changes in barley leaves phenotypes during DILS and DLS progression. **A** Monitoring senescence by RGB imaging. **B- D** fluorescence quenching analysis, three fluorometric parameters were chosen for their sensitivity to the course of senescence, namely **B** ΦPSI- steady- state effective quantum yield of photosystem II, **C** qP- the fraction of open reaction centres of PSII, and **D** Rfd- fluorescence decrease ratio. False colour images of sample plants showing spatio-temporal patterns. The colour scale encoding fluorometric values is given next to the pictures. Time course of fluorescence emission averaged over at least ten plants. 0D – control plants, 4D- day 4 in dark; 7D- day 7 in dark, 10D- day 10 in dark, 5 DBA- 5^th^ day before anthesis, 15 DAA- 15^th^ day after anthesis, 30 DAA- 30^th^ day after anthesis.


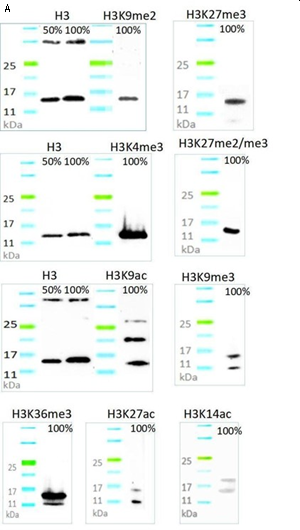


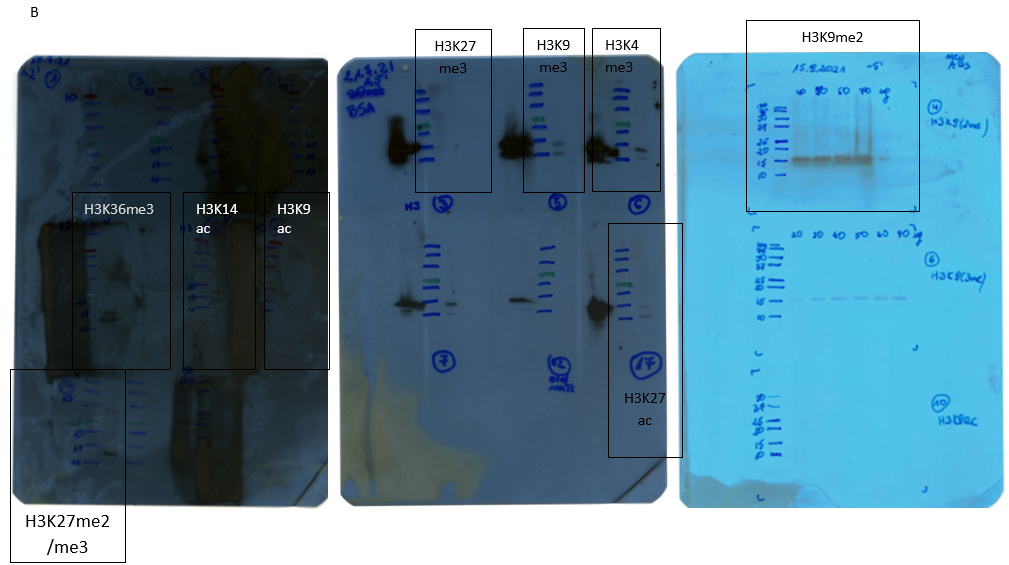


H3K9

ac

H3K14ac

**Fig. S9. Immunodetection of histone 3 (H3) posttranslational modifications in barley leaves**. **A** processed images, **B** non-processed images of western blot analyzes testing commercially available antibodies against H3 posttranslational modifications in barley histone proteins were performed on control leaf material. Set of nine antibodies out of 12 tested showed reactivity against barley proteins. 20-30 ug of sample was loaded on the gel. The detailed list of antibodies is presented in the materials and methods section.


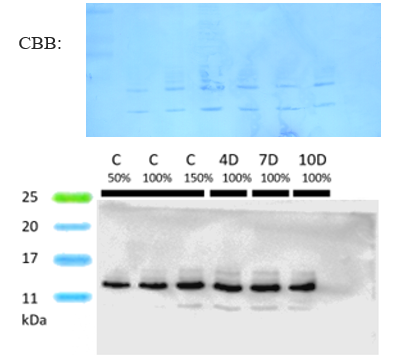


**Fig S10.** Western blot analyses of heterochromatin H3K9me2 histone mark in barley primary leaf during Dark-Induced Leaf Senescence. Control 50%. Control 100%, Control 150%, 4^th^, 7^th^ and 10^th^ day of DILS. Control samples were loaded in three replications in every gel (in 0.5x, 1x, and 2x dilution) to prove the linear range of immunoreaction. The protein standard ladder presents the scale in kDa.


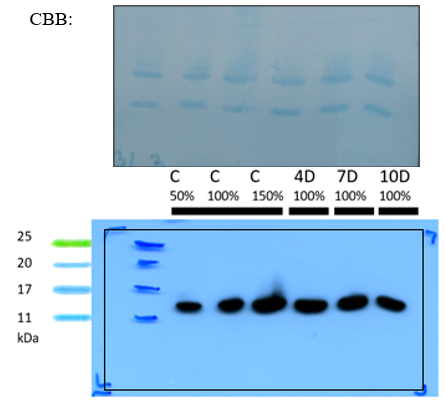


**Fig S11.** Western blot analyses of euchromatin H3K4me3 histone mark in barley primary leaf during Dark-Induced Leaf Senescence. Control 50%. Control 100%, Control 150%, 4^th^, 7^th^ and 10^th^ day of DILS. Control samples were loaded in three replications in every gel (in 0.5x, 1x, and 2x dilution) to prove the linear range of immunoreaction. The protein standard ladder presents the scale in kDa.


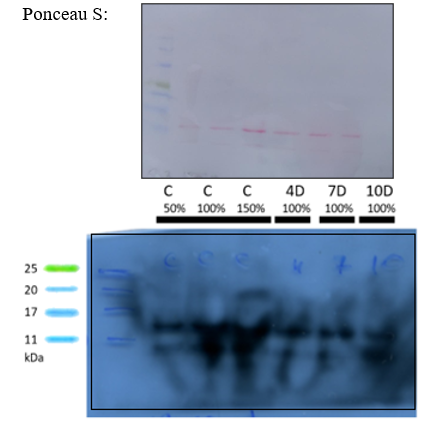


**Fig S12**. Western blot analyses of euchromatin H3K9ac histone mark in barley primary leaf during Dark-Induced Leaf Senescence. Control 50%. Control 100%, Control 150%, 4^th^, 7^th^ and 10^th^ day of DILS. Control (C) samples were loaded in three replications in every gel (in 0.5x, 1x, and 2x dilution) to prove the linear range of immunoreaction. The protein standard ladder presents the scale in kDa.


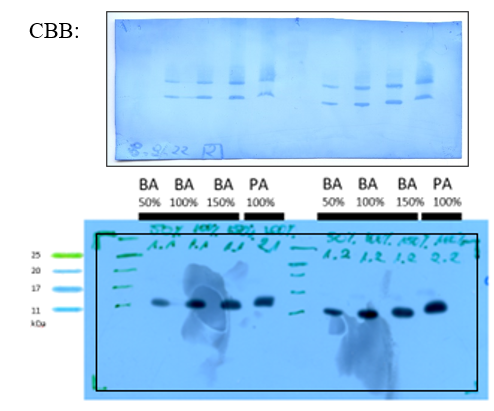


**Fig S13.** Western blot analyses of heterochromatin H3K9me2 histone mark in barley flag leaf during Developmental Leaf Senescence. BA – plants ~5 days before anthesis, PA – plants ~30 days after. The protein standard ladder presents the scale in kDa.


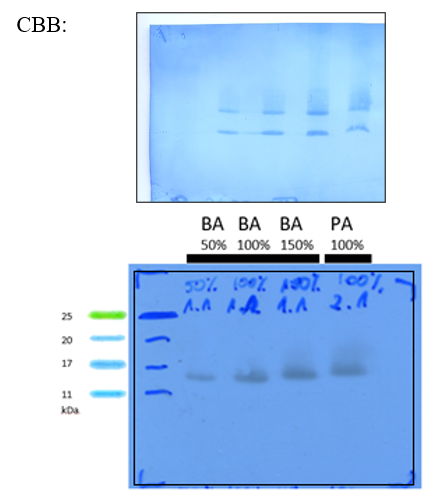


**Fig S14.** Western blot analyses of euchromatin H3K4me3 histone mark in barley flag leaf during Developmental Leaf Senescence. BA – plants ~5 days before anthesis, PA – plants ~30 days after. The protein standard ladder presents the scale in kDa.


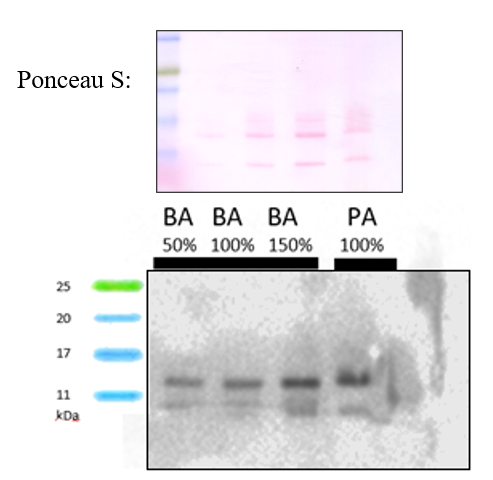


**Fig S15.** Western blot analyses of euchromatin H3K9ac histone mark in barley flag leaf during Developmental Leaf Senescence. BA – plants ~5 days before anthesis, PA – plants ~30 days after. The protein standard ladder presents the scale in kDa.


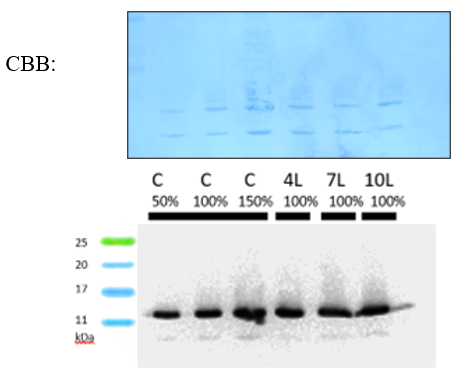


**Fig S16.** Western blot analyses of heterochromatin H3K9me2 histone mark in barley primary leaf in control conditions. C- control plants (plants at day 0), 4L- day 4 in light,7L- day 7 in light, 10L- day 10 in light. Control (C) samples were loaded in three replications in every gel (in 0.5x, 1x, and 2x dilution) to prove the linear range of immunoreaction. The protein standard ladder presents the scale in kDa.


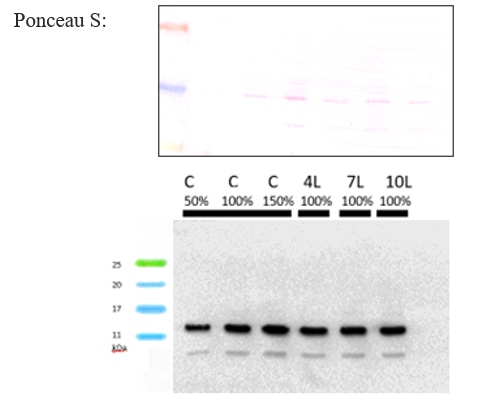


**Fig S17**. Western blot analyses of euchromatin H3K4me3 histone mark in barley primary leaf in control conditions. C- control plants (plants at day 0), 4L- day 4 in light,7L- day 7 in light, 10L- day 10 in light. Control (C) samples were loaded in three replications in every gel (in 0.5x, 1x, and 2x dilution) to prove the linear range of immunoreaction. The protein standard ladder presents the scale in kDa.


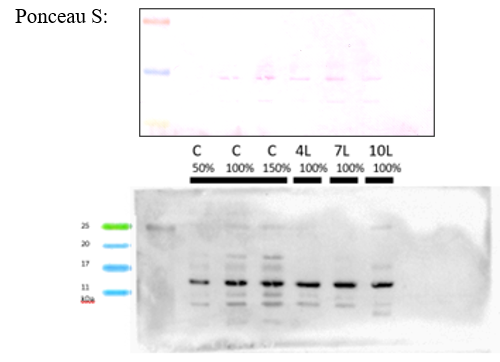


**Fig S18.** Western blot analyses of euchromatin H3K9ac histone mark in barley primary leaf in control conditions. C- control plants (plants at day 0), 4L- day 4 in light,7L- day 7 in light, 10L- day 10 in light. Control (C) samples were loaded in three replications in every gel (in 0.5x, 1x, and 2x dilution) to prove the linear range of immunoreaction. The protein standard ladder presents the scale in kDa.
